# Heavy Prussian Blue Analog with Magnetic Ordering above 400 K

**DOI:** 10.1002/advs.202511285

**Published:** 2025-11-12

**Authors:** Michał Magott, Gabriela Handzlik, Dominik Dzierżek, Alexey Maximenko, Itziar Oyarzabal, Nathan J. Yutronkie, Fabrice Wilhelm, Andrei Rogalev, Dawid Pinkowicz

**Affiliations:** ^1^ Faculty of Chemistry Jagiellonian University Kraków 30‐387 Poland; ^2^ SOLARIS National Synchrotron Radiation Centre Jagiellonian University Kraków 30‐392 Poland; ^3^ BCMaterials Basque Center for Materials, Applications and Nanostructures Leioa 48940 Spain; ^4^ IKERBASQUE Basque Foundation for Science Bilbao 48009 Spain; ^5^ European Synchrotron Radiation Facility (ESRF) Grenoble 38043 France

**Keywords:** coordination polymer, cyanide, magnetic prussian blue analog, molecular magnet, molybdenum, room‐temperature magnetism, vanadium

## Abstract

Molecule‐based magnets hold promise for a variety of applications in information technologies owing to their chemical tunability. This feature can facilitate the integration of desired magnetic properties alongside additional functionalities within a single material. Although numerous cyanido‐bridged assemblies are identified as multifunctional materials at cryogenic temperatures, achieving analogous behavior at room temperature remains a challenge. This study reports a cyanido‐bridged compound, which shows ferrimagnetic ordering with a critical temperature exceeding 400 K. This breakthrough is achieved through the mechanochemical synthesis of vanadium(II)‐hexacyanomolybdate(III) Prussian Blue Analog (PBA) under anhydrous conditions. The ferrimagnetic order is evidenced by SQUID (SQUID = superconducting quantum interference device) magnetometry and X‐ray Magnetic Circular Dichroism (XMCD) spectroscopy. Both techniques unambiguously confirm the antiparallel alignment of vanadium and molybdenum magnetic moments. As a result, hexacyanomolybdate(III) is experimentally established as a viable precursor for the preparation of a new generation of high‐temperature molecule‐based magnets and multifunctional materials.

## Graphical abstract

A cyanido‐bridged vanadium(II)–hexacyanomolybdate(III) Prussian Blue analog synthesized mechanochemically exhibits ferrimagnetic ordering above 400 K. SQUID magnetometry and XMCD experiments confirm antiparallel alignment of V and Mo moments. This establishes hexacyanomolybdate(III) as a robust precursor for high‐temperature molecule‐based magnets, marking a major step toward multifunctional materials operating at ambient conditions.

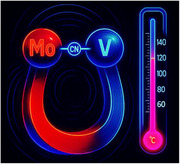

## 1 Introduction

The discovery of ferrimagnetic ordering below 5.6 K in Prussian Blue^[^ ^1^ ^]^ marked an important milestone in the development of new magnetic materials. This catalyzed the emergence of an entire field of molecular magnetism, which initially focused on the exploration of Prussian Blue Analogues (PBAs) based on hexa‐, hepta‐ and octacyanometallates.^[^ ^2^, ^3^, ^4^ ^]^ The in‐depth examination of the magnetic interactions within this family paved the way for the successful design and synthesis of near room‐temperature Cr^II^‐[Cr^III^(CN)_6_] magnets^[^ ^5^, ^6^ ^]^ and above room‐temperature V^II^‐[Cr^III^(CN)_6_] magnets.^[^ ^7^, ^8^ ^]^ The culmination of these efforts through the fine‐tuning of synthetic conditions^[^ ^9^, ^10^, ^11^ ^]^ led to the development of the crystalline KV^II^[Cr^III^(CN)_6_]·2H_2_O with a magnetic ordering temperature (*T*_c_) of 376 K.^[^ ^12^ ^]^ In the realm of molecular magnetism, this material's performance is surpassed only by metal‐radical systems: V^II^(TCNE)_2_, (TCNE = tetracyanoethylene) which shows magnetic ordering temperature exceeding its thermal decomposition (>350 K),^[^ ^13^ ^]^ and the recently discovered Li_0.7_[Cr^II^(pyz)_2_]Cl_0.7_·0.25THF (THF = tetrahydrofuran), with a *T*_c_ of 515 K.^[^ ^14^ ^]^

Low‐density molecular ferrimagnetic materials can offer a plethora of properties. For instance, they can be easily processed into thin films^[^ ^15^, ^16^, ^17^, ^18^ ^]^ which enabled observation of extremely low Gilbert damping.^[^ ^19^, ^20^ ^]^ Structural versatility of these assemblies allowed for the construction of porous frameworks, which exhibit drastic changes in magnetic properties upon adsorption of vapors or gases.^[^ ^21^, ^22^, ^23^, ^24^, ^25^, ^26^ ^]^ Moreover, a variety of multifunctional phenomena were observed for optically transparent magnetic materials based on cyanometallates, including photo‐switching of magnetization,^[^ ^27^, ^28^, ^29^, ^30^, ^31^ ^]^ ultrafast charge transfer,^[^ ^32^, ^33^ ^]^ magneto‐chiral dichroism^[^ ^34^ ^]^ or magnetization‐induced second harmonic generation.^[^ ^35^, ^36^ ^]^ However, efforts to incorporate new chemical components bearing optical functionalities into room‐temperature V^II^‐[Cr^III^(CN)_6_]‐based magnets resulted in the decrease of the observed *T*_c_ below room temperature.^[^ ^37^ ^]^ This shortcoming could be potentially overcome by selecting cyanometallate building blocks that would guarantee magnetic ordering at temperatures even higher than those reported for V^II^‐[Cr^III^(CN)_6_]‐based systems. A replacement of the hexacyanochromate(III) subunits with their heavier hexacyanomolybdate(III) congeners was theoretically predicted to enhance magnetic coupling between metal ions and yield a system with an ordering temperature exceeding 550 K.^[^ ^38^ ^]^ Subsequent experimental work confirmed strong antiferromagnetic exchange coupling constants in discrete clusters [(PY5Me_2_)_4_V^II^_4_Mo^III^(CN)_6_]^5+^ and [V^II^(tmphen)_2_]_3_[Mo^III^(CN)_6_]_2_ (PY5Me_2_ = 2,6‐bis(1,1‐bis(2‐pyridyl)ethyl)pyridine, tmphen = 3,4,7,8‐tetramethyl‐1,10‐phenanthroline).^[^ ^39^, ^40^ ^]^ Despite a prospective precursor in the form of Li_3_[Mo^III^(CN)_6_]·6DMF being reported as early as 2002, the long‐standing challenge to achieve high‐temperature molecular magnets based on hexacyanomolybdate(III) has not been accomplished thus far.^[^ ^41^ ^]^

In this work, we report V^II^‐[Mo^III^(CN)_6_] PBA (**1**) obtained by liquid‐assisted grinding (LAG)^[^ ^42^, ^43^, ^44^ ^]^ and ball milling mechanochemical synthesis. The amorphous compound **1** was characterized by infra‐red (IR) spectroscopy, scanning electron microscopy with energy dispersive X‐ray spectroscopy (SEM‐EDX), X‐ray absorption spectroscopy (XAS) and inductively coupled plasma mass spectrometry (ICP‐MS). The magnetic properties of **1** were investigated by SQUID magnetometry and X‐ray magnetic circular dichroism (XMCD) spectroscopy which revealed an antiparallel alignment of magnetic moments of V^II^ and Mo^III^ to afford a ferrimagnetic order. Finally, **1** exhibits the highest magnetic ordering temperature ever reported for cyanide‐bridged systems, exceeding 400 K. This finding places it as an exceptionally promising candidate for the future development of new, multifunctional molecule‐based magnetic materials operational at room temperature.

## 2 Results and Discussion

### 2.1 Synthesis

The preparation of PBAs typically involves the employment of aqueous solutions.^[^ ^7^, ^8^, ^9^, ^10^, ^11^, ^12^ ^]^ This approach cannot be applied to hexacyanomolybdate(III) precursors due to their tendency to decompose in the presence of protic solvents such as water, methanol or ethanol. To respect the reactive nature of this reagent, our attempts to synthesize **1** were adhered to anhydrous conditions. Thus, we prepared the vanadium complex [V^II^(CH_3_CN)_6_](BF_4_)_2_ (**2**) as a non‐aqueous precursor (see Experimental Section for synthesis details). The choice of [V^II^(CH_3_CN)_6_](BF_4_)_2_ was dictated by the weak coordinating ability of tetrafluoroborate anion. Compound **2** crystallizes in a monoclinic *C*2/*m* space group with two tetrafluoroborate anions per metal center, which indicates the formal +2 oxidation state of the vanadium cation (Table S1 and Figure S1, Supporting Information). The observed V‐N bond lengths of 2.115(2) and 2.127(2) Å are consistent with those of divalent vanadium complexes reported in the CSD database (Figure S2, Supporting Information). Moreover, we have prepared [V^III^(CH_3_CN)_3_Cl_3_]·CH_3_CN (**3**) as a vanadium(III) reference compound for XAS experiments (Table S1 and Figure S3, Supporting Information).

In our initial attempts to synthesize a V^II^‐[Mo^III^(CN)_6_] coordination polymer, Li_3_[Mo^III^(CN)_6_]·6DMF was combined with [V^II^(CH_3_CN)_6_](BF_4_)_2_ (**2**) in a variety of solvents. However, these attempts did not yield any solid products, most likely due to the low solubility of the lithium salt in aprotic solvents such as DMF or acetonitrile.

**Figure 1:**
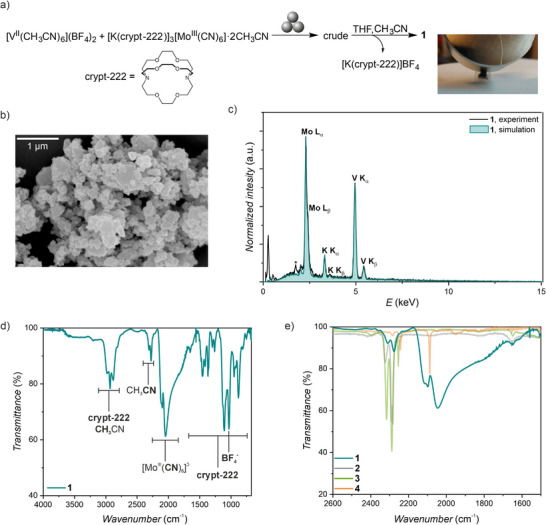
Determination of the chemical composition of **1** | a) Reaction scheme demonstrating mechanochemical preparation of **1** and a photograph of **1** (inside polyethylene bag) being attracted to a neodymium magnet. b) SEM photograph of **1**. c) Experimental EDX spectrum for **1** (black line) and simulation for the K:V:Mo = 0.34:1.37:1 ratio (green line). The signal at 1.75 keV, marked with an asterisk, is attributed to silicon *K*_α_ line and is expected to originate from traces of molecular sieves used for solvent drying. d) Room temperature IR spectrum of **1**. e) Close‐up of nitrile and cyanide stretching region in **1‐4**.

Next, we used [K(crypt‐222)]_3_[Mo^III^(CN)_6_]·2CH_3_CN (**4**) (crypt‐222 = [2.2.2]cryptand), a compound recently reported by us,^[^ ^45^ ^]^ due to its high solubility in acetonitrile. Nevertheless, reactions of acetonitrile solutions of [K(crypt‐222)]_3_[Mo^III^(CN)_6_]·2CH_3_CN and [V^II^(CH_3_CN)_6_](BF_4_)_2_ resulted in the formation of solely colloidal suspensions. Finally, the successful synthesis of solid **1** was achieved using a mechanochemical approach (**Figure** **1**a): mortar and pestle grinding of the two reactants in the presence of a catalytic amount of acetonitrile (LAG method) or ball milling of dry precursors. This facilitated the formation of a reaction mixture containing crystalline [K(crypt‐222)]BF_4_ salt as a byproduct identified by powder X‐ray diffraction (PXRD, Figure S4, Supporting Information). This byproduct was completely washed out with THF and acetonitrile (see Experimental Section for detailed procedure). The remaining dark blue powder (product **1**) was free from crystalline impurities (Figure S5, Supporting Information). The final product is easily attracted to a permanent magnet at room temperature in the inert atmosphere (Figure 1a), but decomposes in several minutes upon exposure to ambient air.

### 2.2 Microscopic, Spectroscopic and Chemical Analysis of **1**

X‐ray diffraction experiments show that **1** is an amorphous material which precludes determination of its crystal structure (Figure S5, Supporting Information). Due to this limitation, we have characterized the morphology of the sample with SEM, its chemical composition with EDX, ICP‐MS and IR spectroscopy, and the oxidation states of metal ions using XAS.

SEM shows that **1** is in the form of submicron particles, which are aggregated into micrometric‐sized grains (Figure 1b). The elemental composition of the isolated product was determined by SEM‐EDX analysis at several sample positions, yielding the average K:V:Mo composition of 0.34(4):1.40(7):1 (Figure 1c). The SEM‐EDX result agrees well with the ICP‐MS analysis, which yields V:Mo = 1.37(2):1.

To further verify the chemical composition of **1**, we recorded its IR spectrum under an argon atmosphere (Figure 1d). The presence of multiple weak transitions in the fingerprint region suggests the incorporation of potassium ions into the structure of **1** in the form of [K(crypt‐222)]^+^ complexes. The presence of tetrafluoroborate counterions is inferred from a band at 1032 cm^−1^, similar to that observed in **2** (Figure S6, Supporting Information). Despite the vacuum treatment of the sample, weak nitrile stretching bands corresponding to the presence of acetonitrile are also observed in the 2200–2320 cm^−1^ range. Most importantly, the main cyanide band of the hexacyanomolybdate(III) moiety at 2088 cm^−1^ which is observed in the starting material **4** is split into three bands with maxima at 2114, 2098 and 2047 cm^−^1 in **1** (Figure 1e). The splitting of the cyanide stretching band is consistent with the presence of both Mo^III^‐CN‐V^II^ cyanide bridges and non‐bridging cyanides in the amorphous **1**.

**Figure 2:**
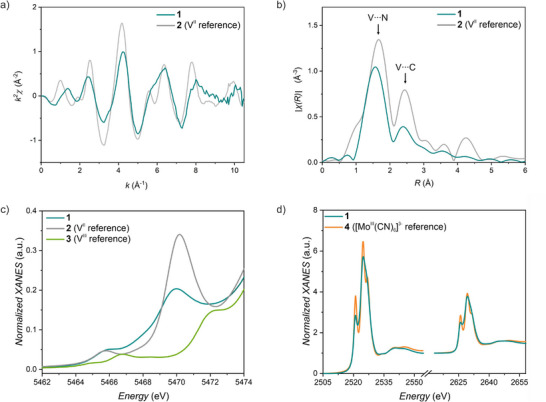
XAS analysis of coordination environment and oxidation state of **1** | a) *k* ^2^‐weighted EXAFS spectra at the V *K*‐edge for **1** and **2**. b) Fourier‐transform (FT) EXAFS spectra for **1** and **2**. c) Pre‐edge transitions in the vanadium *K*‐edge spectrum of **1**‐**3**. d) Comparison of the molybdenum *L*_2,3_‐edges spectra of **1** and **4**.

Further evidence for the presence of V^II^‐NC linkages comes from the analysis of the extended X‐ray absorption fine structure (EXAFS), observed in the XAS spectra of **1** and **2** recorded in the transmission mode (Figure S7, Supporting Information). Overlaying the *k* ^2^‐weighted EXAFS spectra (**Figure** **2**a) of **1** and the [V^II^(NCCH_3_)_6_]^2+^ precursor **2** reveals the similarity of the vanadium coordination sphere in both compounds. However, the general damping and broadening of the EXAFS oscillations confirms the formation of a new vanadium‐based phase, consistent with the PXRD and IR results. The observed changes also suggest non‐uniform geometry of vanadium centers in **1**. The Fourier transform of the spectrum reveals two EXAFS peaks at radial distances *R* = 1.66 Å and *R* = 2.42 Å for **2** (Figure 2b, grey line). These peaks can be attributed to the scattering from nitrogen and carbon atoms located 2.12 Å and 3.24 Å from the vanadium center in the crystal structure, respectively. Similar peaks are observed for 1 at *R* = 1.56 Å at *R* = 2.39 Å (Figure 2b, green line), which confirms the presence of vanadium cations coordinated by ‐NC ligands in both compounds. For a detailed discussion and analysis of EXAFS in **1** and **2**, please see the relevant section in Supporting Information as well as Figure S8 and Table S2 (Supporting Information) therein.

The oxidation states of vanadium and molybdenum in **1** were investigated using XAS at the vanadium *K*‐edge and molybdenum *L*_2,3_‐edges (Figure 2c‐d) detected using total fluorescence yield detection mode. For the former, the energies of the pre‐edge transitions involving the 1*s* → 3*d* excitations can be used as a fingerprint of the oxidation state of vanadium. Accordingly, the spectrum of **1** was compared with [V^II^(CH_3_CN)_6_](BF_4_)_2_ (**2**) and [V^III^Cl_3_(CH_3_CN)_3_]·CH_3_CN (**3**), which serve as reference compounds for octahedral V(II) and V(III), respectively. In the case of **1**, two main transitions are located at 5466.1 and 5470.0 eV, similar to 5465.8 and 5470.2 eV features observed in **2** (Figure 2c; Figure S9, Supporting Information). On the contrary, the vanadium(III) compound **3** exhibits spectral structures at 5465.0, 5466.8 and 5472.3 eV, which are clearly different from peaks observed in **1** and **2**. This indicates that vanadium in **1** has the formal +2 oxidation state.

In contrast to the vanadium *K*‐edge, the energy positions of the so called “white lines” (resonant 2*p* → 4*d* transitions) observed at the Mo *L*_2,3_‐edges cannot be directly related to the oxidation state of the metal ion. However, the integrated intensity of the white lines sum up over two edges is directly proportional to the number of 4*d* holes in Mo.^[^ ^46^ ^]^ The fine structure of the white lines is due to the ligand field splitting, reflecting coordination geometry of the metal ion. Despite the apparent difference in the spectral shapes for **1** and **4** (Figure 2d), the integrated intensities of the white lines were found to be identical within 3%. These results unambiguously confirm the +3 oxidation state of Mo in **1**. The fine structure in **1** is broadened due to the amorphous nature of the sample. Considering the determined oxidation states of vanadium and molybdenum in **1**, and the presence of the [K(crypt‐222)]^+^ moiety in its structure, the amount of 0.08 tetrafluoroborate anion per Mo was estimated based on the charge balance. In conclusion, the above analysis asserts the formulation of **1** as {[K(crypt‐222)]_0.34_V^II^_1.37_[Mo^III^(CN)_6_](BF_4_)_0.08_·xCH_3_CN}*_n_*.

### 2.3 Magnetic Properties of **1**

**Figure 3:**
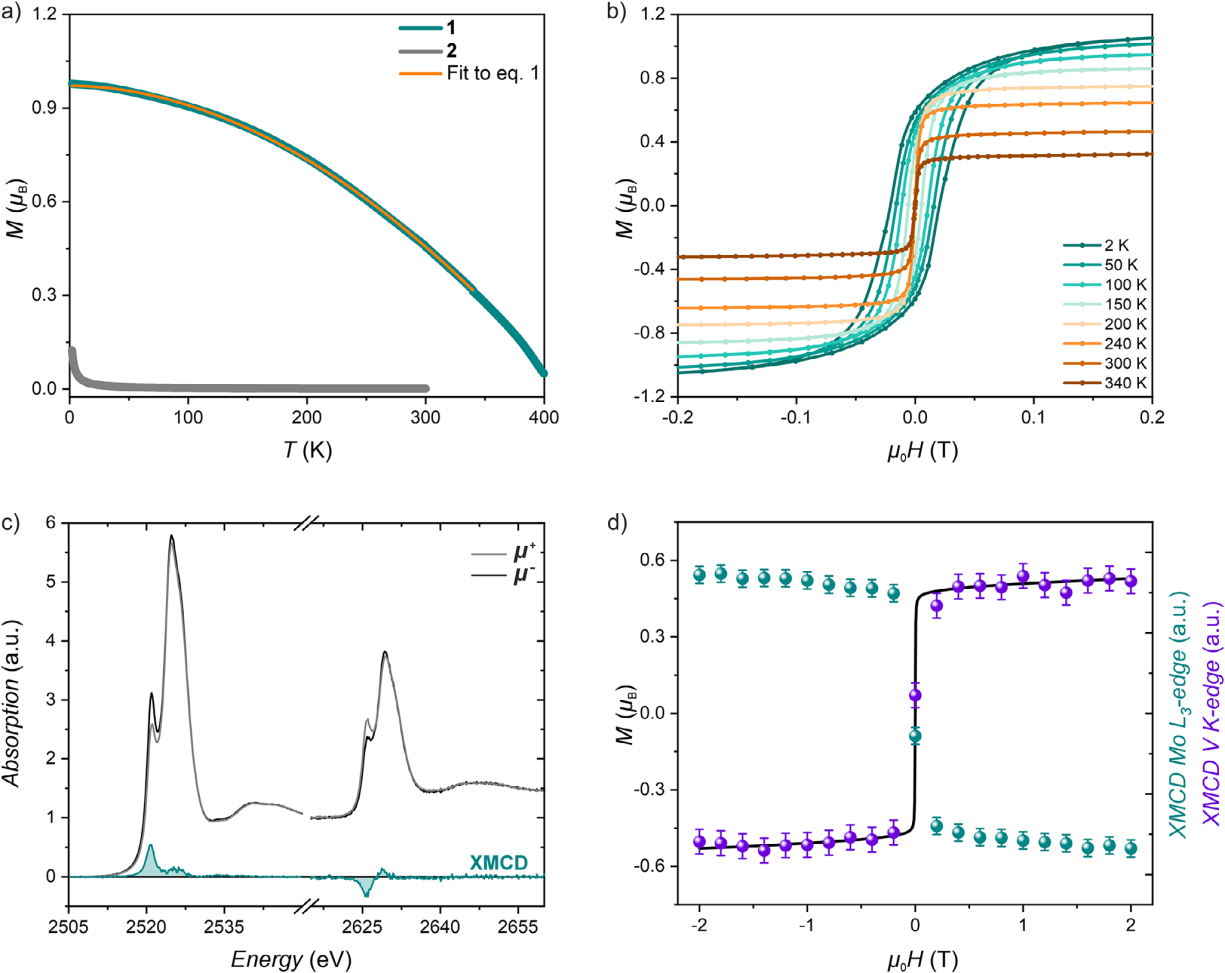
Room temperature magnetic behavior of **1** | a) Temperature dependence of magnetization (*M*) recorded for **1** and **2** under an applied dc magnetic field of 0.1 T b) Variable‐temperature magnetic hysteresis loops recorded for **1**. c) Room temperature Mo *L*_2,3_‐edge XAS spectra recorded for **1** at the opposite circular polarizations of incoming X‐rays (black and grey lines), and the corresponding XMCD signals (green lines). d) Field dependence of the macroscopic magnetization recorded for **1** at 295 K (black line), compared with the field‐dependence of XMCD signals (XMCD signals were rescaled to match macroscopic magnetization data at 2 T) obtained for **1** at the Mo *L*_3_‐edge and V *K*‐edge at 295 K (green and purple points; Mo signal was inverted to reflect antiparallel orientation of the magnetic moment of Mo as deduced from magneto‐optic sum rules).

The molar magnetization (*M*) of **1** under an applied field of 0.1 T reaches 0.98 *μ*_B_ at 2.0 K (**Figure** **3**a, green line). The *M*(*T*) curves recorded during subsequent heating and cooling cycles overlap up to 340 K (Figure S10, Supporting Information), where the irreversible thermal decomposition of the sample begins, which is consistent with the TGA results. Nevertheless, the magnetization of **1** amounts to 0.32 *μ*_B_ at 340 K and 0.05 *μ*_B_ at 400 K, which is two orders of magnitude higher than the paramagnetic response of the starting material **2** (Figure 3a, grey line; see Figure S11 (Supporting Information) for the magnetic characterization of **2**). To estimate the critical temperature of the magnetic ordering, the magnetization in the 2–340 K range was fitted using the Bloch's equation:^[^ ^47^ ^]^

(1) $$M\left(T\right)=M_{0}\cdot \left[1-{\left(T/T_{c}\right)}^{\alpha }\right]$$

A classical formulation of Bloch's law with *α* = 3/2 yields a *T*_c_ of 454 K but the quality of the fit of the experimental data is unsatisfactory (*R* ^2^ = 0.9911, Figure S12, Supporting Information). Using the empirical model with *α* exponent as a free parameter affords much better agreement with *α* = 1.927(1) and *T*_c_ of 417 K (*R* ^2^ = 0.9998, Figure 3a). The increased value of *α* was also reported for the amorphous V(TCNE)_2_ molecular magnet^[^ ^48^ ^]^ and metal oxide magnetic nanoparticles.^[^ ^49^, ^50^ ^]^ Unfortunately, direct calculation of the superexchange coupling in **1** is hardly possible due to the amorphous nature of the sample. Nevertheless, a rough estimation can be obtained by assuming *z*_V_ = 4 nearest neighbors for vanadium(II) cations and *z*_Mo_ = 6 for hexacyanomolybdate(III) sites, using the molecular field approach:^[^ ^51^ ^]^

(2) $$T_{c}=\frac{2\sqrt{z_{V}z_{Mo}}\left|J_{V-Mo}\right|\sqrt{xS_{V}\left(S_{V}+1\right)S_{Mo}\left(S_{Mo}+1\right)}}{3k_{B}}$$

For *T*_c_ = 417 K and x = 1/1.37 = 0.73, the obtained superexchange coupling is equal to *J*_VMo_ = −28 cm^−1^. This value is considerably smaller than *J*_V‐Mo_ = −61 cm^−1^ determined for V_4_Mo clusters^[^ ^40^ ^]^ and *J*_V‐Mo_ = −114 cm^−1^ for V_3_Mo_2_ clusters.^[^ ^39^ ^]^ The discrepancy is likely associated with either an overestimation of the number of V^II^‐NC‐Mo^III^ bridges (*z*_V_ and *z*_Mo_), or their non‐linearity as a consequence of the structural disorder in the amorphous state.^[^ ^10^, ^11^ ^]^

Compound **1** exhibits behavior consistent with that of a soft magnet, with a maximum magnetic coercivity (*H*_c_) equal to *μ*_0_ *H*_c_ = 0.02 T at 2 K (Figure 3b). Accordingly, the magnetization is readily saturated, with the magnetization value of 1.11 *μ*_B_ at 1 T already reaching 96% of the saturation value of 1.16 *μ*_B_ at 7 T (Figure S13, Supporting Information). The experimental value indicates the antiparallel alignment of magnetic moments on vanadium and molybdenum, as deduced from the following formula:

(3) $$\begin{array}{ccc} M_{\mathrm{sat}} & = & x_{V}\cdot g_{V}\cdot S_{V}-\;x_{\mathrm{Mo}}\cdot g_{\mathrm{Mo}}\cdot S_{\mathrm{Mo}}=1.37\cdot 1.97\cdot 3/2\;\\ & & -\;1.97\cdot 3/2=1.09\;\mu_{B} \end{array}$$

where *g*_V_ = 1.97 and *g*_Mo_ = 1.97 were assumed to be the same as in the precursors: **2** and **4**,^[^ ^45^ ^]^ respectively. Upon heating, the width of the magnetization hysteresis is decreasing, and above 200 K the coercivity becomes smaller than the remanent field of the superconducting magnet of the SQUID magnetometer (ca. 0.003 T). Nonetheless, the sigmoidal shape of the magnetization curve typical for soft magnets (unlike the paramagnetic magnetization curve) is observed up to 340 K.

The element‐specific magnetic moments of **1** were investigated using the XMCD spectroscopy. The XMCD spectra collected for **1** at room temperature (*T* = 295 K) and under an applied magnetic field of 2 T show intense signals at both Mo *L*_2,3_‐edges (Figure 3c). Their signs evidence antiparallel orientation of the magnetic moment of molybdenum with respect to the external magnetic field. This confirms ferrimagnetic ordering in **1** at 295 K as inferred from macroscopic magnetization measurements. Spin and orbital magnetic moments carried by molybdenum ions are quantitatively analyzed using the magneto‐optical sum rules^[^ ^52^, ^53^ ^]^ (see Experimental Section for details). This analysis reveals the spin contribution to the magnetic moment of *M*_S_ = −1.08 *μ*_B_ and the orbital contribution of *M*_L_ = −0.04 *μ*_B_. The spin‐dominated magnetism of molybdenum(III) in **1** is in agreement with previous results for the hexacyanomolybdate(III).^[^ ^45^ ^]^ The molybdenum magnetic moment determined from magneto‐optical sum rules *M* ^Mo^ = *M*_S_ + *M*_L_ = −1.12 *μ*_B_ is in good agreement with the value of −1.25 *μ*_B_ obtained by scaling macroscopic magnetization (+0.47 *μ*_B_ at 295 K). Considering the positive macroscopic magnetization of the sample, the magnetic moment of vanadium must be aligned parallel to the magnetic field. Unfortunately, XMCD spectra at *K*‐edge cannot be analyzed quantitatively. However, the derivative‐shaped signal recorded for the vanadium *K*‐edge spectrum collected for **1** at room temperature (*T* = 295 K) and under applied field of 2 T (Figure S14, Supporting Information) is consistent with the previous observations in PBAs.^[^ ^8^, ^54^, ^55^ ^]^

Overall, the element‐selective XMCD analysis for **1** clearly demonstrates that the magnetic moments of V^II^ and Mo^III^ are antiparallel to each other, thereby confirming the antiferromagnetic nature of the superexchange interactions and the ferrimagnetic nature of the sample. To get further insight into magnetism of **1**, we examined the field dependence of the maxima of the XMCD signals at the Mo *L*_3_‐edge and V *K*‐edge (Figure 3d). The element‐specific XMCD magnetization curves for both edges are found to scale in the similar manner as the macroscopically measured magnetization of **1** recorded using *dc* magnetometry (black line in Figure 3d). This confirms that the observed macroscopic magnetic properties of **1** are due to its intrinsic ferrimagnetic ordering.

## 3 Conclusion

In conclusion, we have demonstrated that the heavy Prussian Blue Analog {[K(crypt‐222)]_0.34_V^II^_1.37_[Mo^III^(CN)_6_](BF_4_)_0.08_·xCH_3_CN}*_n_* (**1**), synthesized using the hexacyanomolybdate(III) anion, exhibits magnetic ordering at a temperature exceeding that of all previously reported cyanide‐bridged systems. The ferrimagnetic order persists above 400 K despite the amorphous nature of the solid. The successful incorporation of various organic ligands or cations into the structure of V^II^‐[Mo^III^(CN)_6_] assemblies may, in the future, facilitate the construction of room‐temperature multifunctional magnets.

## 4 Experimental Section

### General Considerations

All manipulations were performed under an argon gas atmosphere inside the Inert PureLab HE glovebox (O_2_ < 0.1 ppm and H_2_O < 0.5 ppm). All solvents used in this study were of HPLC grade and were additionally dried under argon using the Inert PureSolv EN7 solvent purification system and stored over 3 Å molecular sieves for at least 24 h before use. VCl_3_(THF)_3_ and K_4_[Mo^III^(CN)_7_]·2H_2_O were prepared according to the reported procedures.^[^ ^56^, ^57^, ^58^ ^]^ All the other reagents were purchased from commercial suppliers and used without further purification.

### Preparation of {[K(crypt‐222)]_0.34_V^II^_1.37_[Mo^III^(CN)_6_](BF_4_)_0.08_·xCH_3_CN}_n_ (**1**)—Liquid‐Assisted Grinding (LAG) Method

[V^II^(CH_3_CN)_6_](BF_4_)_2_ (47 mg, 0.1 mmol) and [K(crypt‐222)]_3_[Mo^III^(CN)_6_]·2CH_3_CN (158 mg, 0.1 mmol) were ground in an agate mortar. Then 150 µL acetonitrile was added to the powder, and grinding was continued for 15 min. After this time, the solid mixture was transferred to a Schlenk flask and heated to 50 °C under vacuum for 1 h. The obtained powder was suspended in 20 mL THF for 1 h followed by the filtration using 1.0 µm pore size PTFE membrane. The resulting dark blue solid was washed with 3 x 20 mL acetonitrile, 20 mL THF and 20 mL diethyl ether, and then dried at 50 °C under vacuum for 1 h. The final product was obtained in 80% yield (30 mg), based on [V^II^(CH_3_CN)_6_](BF_4_)_2_. The elemental composition was determined by SEM‐EDX (average molar composition: K:V:Mo = 0.34(4):1.40(7):1) and ICP‐MS (mass percentage: V, 10.63(7)%; Mo = 14.61(9)%; molar ratio: V:Mo = 1.37(2):1), as described in the main text.

### Preparation of {[K(crypt‐222)]_0.34_V^II^_1.37_[Mo^III^(CN)_6_](BF_4_)_0.08_·xCH_3_CN}_n_ (**1**)—Ball Milling Method

[V^II^(CH_3_CN)_6_](BF_4_)_2_ (82 mg, 0.175 mmol) and [K(crypt‐222)]_3_[Mo^III^(CN)_6_]·2CH_3_CN (276 mg, 0.175 mmol) were loaded into a 12 mL agate grinding bowl along with 50 agate grinding balls (5 mm diameter). The grinding bowl was then fitted with a Teflon flat seal and an agate lid. The narrow gap between the lid and the bowl was filled with an acid‐free silicone sealant (Victor Reinz Reinzosil), after which the bowl was tightened with a G‐clamp. After allowing the sealant set for 12 h, the grinding bowl was moved outside the glovebox and inserted into a planetary ball mill (Fritsch PULVERISETTE 7 classic line). The as‐prepared setup was subjected to 36 cycles of milling at 800 rpm (2 min each), with 3‐min pauses in between to avoid heating the reaction mixture above 50 °C. Once milling had finished, the grinding bowl was returned to the glovebox, the silicone sealing was cut open, and the grinding balls with reaction mixture were transferred to a glass jar. Then, 20 mL THF were added, and the reaction mixture was left for 2 h. The suspension was decanted from the agate balls and then filtered using 1.0 µm pore size PTFE membrane. The resulting dark blue solid was washed with 20 mL acetonitrile and then dried at 50 °C under vacuum for 1 h. The final product was obtained with 45% yield (30 mg), based on [V^II^(CH_3_CN)_6_](BF_4_)_2_ and it shows magnetic behavior identical to that obtained via LAG method.

### Preparation of [V^II^(CH_3_CN)_6_](BF_4_)_2_ (**2**)

VCl_3_(THF)_3_ (0.75 g, 2.0 mmol) dissolved in 8 mL acetonitrile was combined with a solution of AgBF_4_ (1.22 g, 6.27 mmol; Sigma‐Aldrich, 98%) in 8 mL acetonitrile. After 15 min of constant stirring, solution was filtered through sintered glass funnel (G3). The filtrate was mixed with CoCp_2_ (0.40 g, 2.1 mmol; Sigma‐Aldrich reagent grade) and stirred for another 30 min. The resulting mixture was filtered through sintered glass funnel (G4) and the filtrate was treated with 70 mL diethyl ether. The green precipitate was collected by filtration, dissolved in 7 mL acetonitrile and subjected to slow diffusion of diethyl ether vapors. After 24 h compound **2** crystallized as dark green‐blue crystals, which were collected by filtration and washed with diethyl ether. The final product was obtained in 59% yield (0.56 g), based on VCl_3_(THF)_3_. The purity of the compound was checked by powder X‐ray diffraction, with the experimental pattern (Figure S15, Supporting Information) matching perfectly the simulated one from the scXRD structural model obtained at 180 K (crystal structure was deposited under CCDC 2417011).

### Preparation of [V^III^(CH_3_CN)_3_Cl_3_]·CH_3_CN (**3**)

Compound **3** was prepared by slow diffusion of diethyl ether vapor onto saturated acetonitrile solution of VCl_3_(THF)_3_ at room temperature. After 2 days compound **3** crystallized as light green crystals suitable for single‐crystal X‐ray diffraction, which was performed at 180 K (crystal structure was deposited under CCDC 2417010).

### Preparation of [K(crypt‐222)]_3_[Mo^III^(CN)_6_]·2CH_3_CN (**4**)^[^ ^45^ ^]^

K_4_[Mo^III^(CN)_7_]·2H_2_O (0.24 g, 0.51 mmol) was dissolved in 7 mL acetonitrile in the presence of [2.2.2]cryptand (crypt‐222; 0.9 g, 2.39 mmol; Merck, ≥99%). The yellow suspension was irradiated with white light (*P* = 150 W, Photonic LED‐Light‐Source F3000) until it became an almost colorless solution. Then it was subjected to slow diffusion of diethyl ether vapor for two days, resulting in the precipitation of colorless plate crystals, which were collected by vacuum filtration. The final product (0.7 g) was obtained in 87% yield based on K_4_[Mo^III^(CN)_7_]·2H_2_O. The purity of the compound was checked by powder X‐ray diffraction, with the experimental pattern (Figure S16, Supporting Information) matching perfectly the simulated one from the scXRD structural model obtained at 293 K (crystal structure was deposited under CCDC 2352266).

### Single Crystal X‐Ray Diffraction

The scXRD experiment for **2** (CCDC 2417011) was performed using Bruker D8 Quest Eco diffractometer (sealed tube Mo *K*_α_ radiation, Triumph monochromator). The scXRD experiment for **3** (CCDC 2417010) was performed using Bruker D8 Venture diffractometer (Mo *K*_α_ INCOATEC IµS 3.0 microfocus sealed tube radiation source, Helios optics). Single crystals were transferred directly from the mother liquor into the NVH immersion oil (Cargille) to avoid loss of crystallization solvent and prevent them from decomposition in air. Absorption corrections, data reduction, and unit cell refinements were performed using SADABS and SAINT programs included in the Apex3 suite. Structures were solved using intrinsic phasing and refined anisotropically using weighted full‐matrix least‐squares on *F* ^2^.^[^ ^59^, ^60^, ^61^ ^]^ Hydrogen atoms of ligands were placed in calculated positions and refined as riding on the parent atoms. Structural diagrams were prepared using Mercury CSD 2020.3.0.^[^ ^62^ ^]^

### Powder X‐Ray Diffraction

PXRD patterns were collected using Bruker D8 Advance diffractometer (Cu Kα radiation) at room temperature from samples loaded into sealed glass capillaries (0.5 mm in diameter).

### Magnetic Measurements

Magnetic data were collected using Quantum Design MPMS‐3 Evercool magnetometer. Powder sample of **1** was sealed inside glass tube with a small amount of paraffin oil and then inserted into plastic straw. Powder sample of **2** was placed inside polyethylene bag, sealed and attached with Kapton tape to the quartz sample holder. The measurement data were corrected for diamagnetism of the samples.

### Infrared (IR) Spectroscopy

IR spectra were recorded using a Nicolet iN10 MX FT‐IR microscope in transmission mode (a small amount of powdered sample was spread using a roller on a BaF_2_ single crystal substrate). To prevent sample decomposition in contact with air, they were loaded into a Linkam THMS350V temperature‐controlled stage inside Ar‐filled glovebox.

### Scanning Electron Microscopy (SEM) and Energy‐Dispersive X‐Ray Spectroscopy (EDX)

The SEM photograph presented in Figure 1b was collected using a HITACHI S‐4700 microscope at an accelerating voltage of 20 kV, for a sample of **1** coated with gold. SEM‐EDX microanalysis was performed on **1** coated with carbon, using a NORAN Vantage system and a NanoTrace SiLi detector. The NSS Microanalysis System software was employed for the determination of sample composition, and NIST DTSA‐II software^[^ ^63^, ^64^ ^]^ was used to simulate the spectrum demonstrated in Figure 1c.

### Inductively Coupled Plasma Mass Spectrometry (ICP‐MS) Analysis

ICP‐MS analysis was performed using a PerkinElmer ELAN DRC‐e spectrometer on a sample of **1** (ca. 3 mg) dissolved on air in a 25 mL saturated water solution of Na_2_EDTA.

### Extended X‐Ray Absorption Fine Structure (EXAFS)

X‐ray absorption spectra for the purpose of EXAFS analysis were recorded at the ASTRA beamline of the SOLARIS Synchrotron in Kraków, Poland.^[^ ^65^, ^66^ ^]^ Spectra were recorded using a photon beam produced by a double‐bend achromatic 1.3 T bending magnet with a critical energy of ≈2 keV. The beam was monochromatized using a Si(111) crystal pair, resulting in a beam size of 7 x 1 mm at the sample position. The spectra were acquired in transmission mode using ionization chambers and a sample chamber filled with nitrogen gas at 400 torr. A vanadium foil from Exafs Company (USA) served as a reference. Inside Ar‐filled glovebox, powdered samples were spread as thin layers on polypropylene foil and fixed to a sample holder (a metallic frame) using double‐sided adhesive tape. The as‐prepared samples were sealed inside polyethylene bag. The processing of experimental EXAFS spectra was done using the ATHENA software.^[^ ^67^ ^]^ No corrections for the phase and amplitude of backscattering atoms were applied.

### X‐Ray Absorption Spectroscopy (XAS) and X‐Ray Magnetic Circular Dichroism (XMCD)

X‐ray absorption spectra at the Mo *L*_2,3_‐edges and V *K*‐edge were recorded at the ID12 beamline (ESRF, The European Synchrotron Radiation Facility).^[^ ^68^ ^]^ Powdered samples mixed with mineral oil were mounted inside Ar‐filled glovebox into vacuum tight sample holders covered with polyethylene terephthalate film. The spectra were measured at room temperature using total fluorescence yield detection mode and were subsequently corrected for re‐absorption effects. The X‐ray Magnetic Circular Dichroism (XMCD) spectra were obtained as the difference between two consecutive XAS spectra recorded with opposite photon helicities and corrected for the incomplete circular polarization rate. To ensure the absence of experimental artefacts the measurements were systematically performed for both magnetic field directions. The normalized spectra were analyzed using the magneto‐optical sum rules for Mo *L*_2_ and *L*_3_ absorption edges^[^ ^52^ ^]^ to deduce spin magnetic moment (*M*_S_ = ‐2<*S*_z_>*μ*_B_ where <*S*_z_>=<*S*_eff_>‐7/2<*T*_z_>) and orbital magnetic moment (*M*_L_ = <*L*_z_>*μ*_B_):

(4) $$\langle L_{z}\rangle =\frac{2\langle n_{h}\rangle }{3}\cdot \frac{I_{L_{3}}^{\mathrm{XMCD}}+I_{L_{2}}^{\mathrm{XMCD}}}{I_{L_{3}}^{\mathrm{XAS}}+I_{L_{2}}^{\mathrm{XAS}}}$$

(5) $$\langle S_{eff}\rangle =\frac{\langle n_{h}\rangle }{2}\cdot \frac{I_{L_{3}}^{XMCD}-2I_{L_{2}}^{XMCD}}{I_{L_{3}}^{XAS}+I_{L_{2}}^{XAS}}$$

The average value of the *z*‐component of the magnetic dipole operator (*T*_z_) in a bimetallic **1** cannot be directly determined by experiment. In order to estimate the *T*_z_ contribution, XMCD measurements were performed at Mo *L*_2,3_‐edges in the paramagnetic [Mo^III^(CN)_6_]^3−^ precursor **4** (Figure S17, Supporting Information), in which macroscopic magnetization determined by SQUID magnetometry (*M*_total_ = *M*_S_ + M*_L_*) arises solely from the molybdenum(III) ions. The *T*_z_/*M*_s,eff_ ratio in [Mo^III^(CN)_6_]^3−^, elucidated by comparison of macroscopic *M*_s_ with that determined from magneto‐optical sum rules, was assumed to be the same in **1** and **4**.

The element‐selective magnetization curves depicted in Figure 3d were recorded by monitoring the maximum of XMCD signal (at 2520.7 eV for Mo, at 5477.4 eV for V) as a function of an applied magnetic field.

### Thermogravimetric Analysis (TGA)

TGA for compound **1** (Figure S18, Supporting Information) was recorded using a NETZSCH TG 209 F1 Libra thermogravimeter under a flow of dry nitrogen gas of 20 mL·min^−1^ and a temperature scanning rate of 2 K·min^−1^.

## Conflict of Interest

The authors declare no conflict of interest.

## Supporting information



Supporting Information

Supplemental Movie 1

## Data Availability

The data that support the findings of this study are available from the corresponding author upon reasonable request.
