# Supplementary material for: Heavy Prussian Blue Analog with Magnetic Ordering above 400 K
Source: Adv Sci (Weinh). 2025 Nov 12;13(5):e11285. doi: 10.1002/advs.202511285 (PMC12850227; doi:10.1002/advs.202511285)
Supplement: Supplementary file 1 — Supporting Information [file ADVS-13-e11285-s002.docx]

Supporting Information

Heavy Prussian Blue Analog with Magnetic Ordering Above 400 K

Michał Magott,* Gabriela Handzlik, Dominik Dzierżek, Alexey Maximenko, Itziar Oyarzabal, Nathan J. Yutronkie, Fabrice Wilhelm, Andrei Rogalev, Dawid Pinkowicz*

**Table S1.** Crystal structure parameters for **2** and **3.**

| compound | | **2** | **3** |
| --- | --- | --- | --- |
| CCDC no. | | 2417011 | 2417010 |
| method | | single-crystal XRD | single-crystal XRD |
| formula | | C_12_H_18_N_6_V·2(BF_4_) | C_16_H_24_Cl_6_N_8_V_2_ |
| formula weight (g·mol^-1^) | | 470.88 | 643.01 |
| *T* (K) | | 180(2) | 180(2) |
| *λ* (Å) | | 0.71073 (Mo Kα) | 0.71073 (Mo Kα) |
| crystal system | | Monoclinic | Triclinic |
| space group | | *C*2/*m* | *P*$\bar{1}$ |
| unit cell | ***a* (Å)** | 12.0918(8) | 8.643(4) |
|  | ***b* (Å)** | 17.2489(14) | 12.207(10) |
|  | ***c* (Å)** | 5.7035(4) | 14.854(10) |
|  | **α (deg)** | 90 | 83.745(12) |
|  | **β (deg)** | 105.088(3) | 87.51(2) |
|  | **γ (deg)** | 90 | 75.013(16) |
| *V* (Å^3^) | | 1148.57(15) | 1504.7(17) |
| *Z* | | 2 | 2 |
| calculated density (g·cm^-3^) | | 1.362 | 1.419 |
| absorption coefficient (mm^-1^) | | 0.50 | 1.17 |
| *F*(000) | | 474 | 648 |
| θ range (deg) | | 3.8-26.8 | 2.1-28.3 |
| collected reflections | | 4733 | 21448 |
| *R*_int_ | | 0.027 | 0.069 |
| completeness (%] | | 98.7 | 98.6 |
| data/restraints/parameters | | 1260/1/94 | 7435/0/297 |
| GOF on *F*^2^ | | 1.13 | 1.06 |
| final *R* indices | | *R*_1_ = 0.042 [*I* > 2σ(*I*)]  w*R*_2_ = 0.111 (all data) | *R*_1_ = 0.053 [*I* > 2σ(*I*)]  w*R*_2_ = 0.121 (all data) |
| largest diff peak and hole | | 0.34 and -0.28 e·Å^-3^ | 0.41 and -0.42 e·Å^-3^ |
| crystal size  (mm x mm x mm) | | 0.30 x 0.08 x 0.06 | 0.21 x 0.18 x 0.08 |


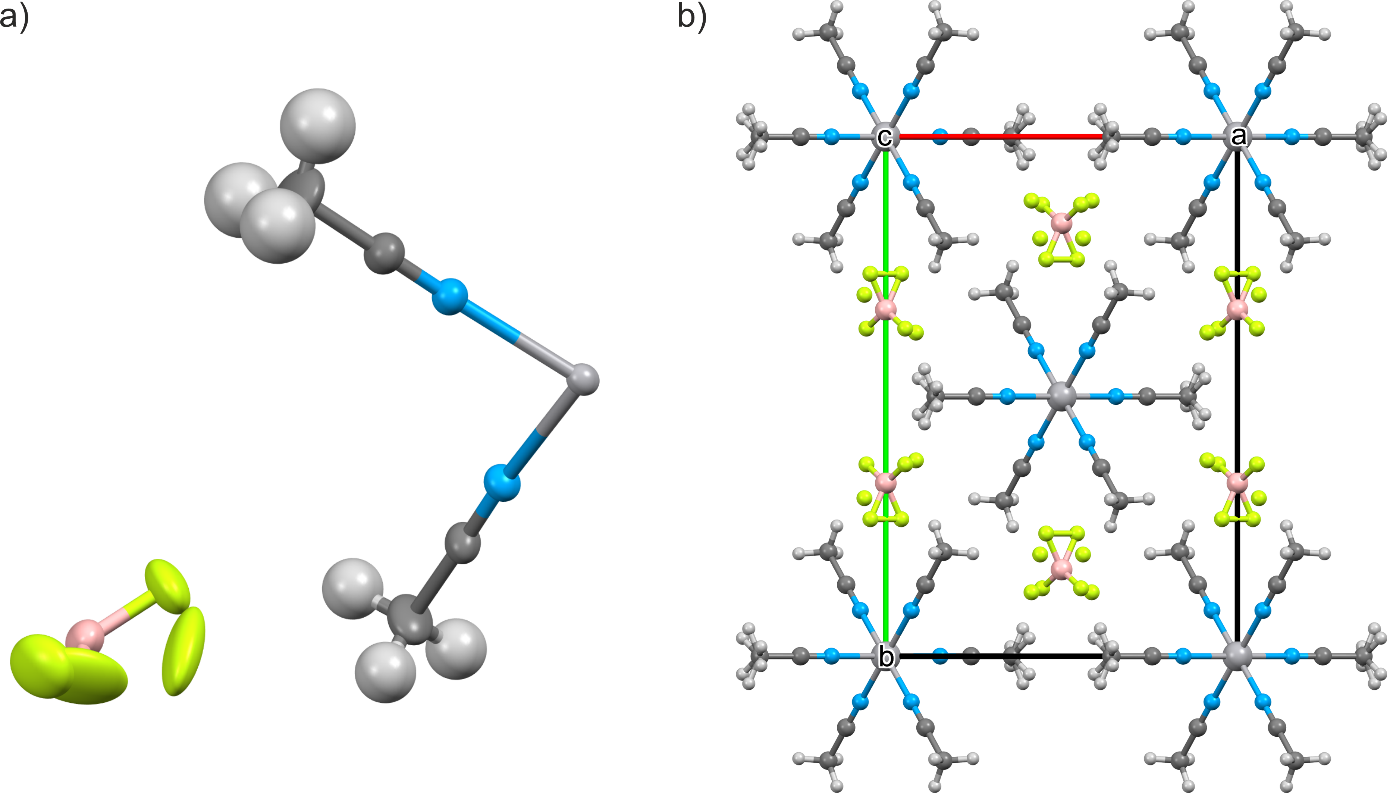


**Figure S1.** a) Asymmetric unit of **2**. Ellipsoids are depicted at 30% probability level. b) Unit cell of **2** along crystallographic *c* axis. V – grey, F – green, N – blue, C – dark grey, B – pink, H – white.


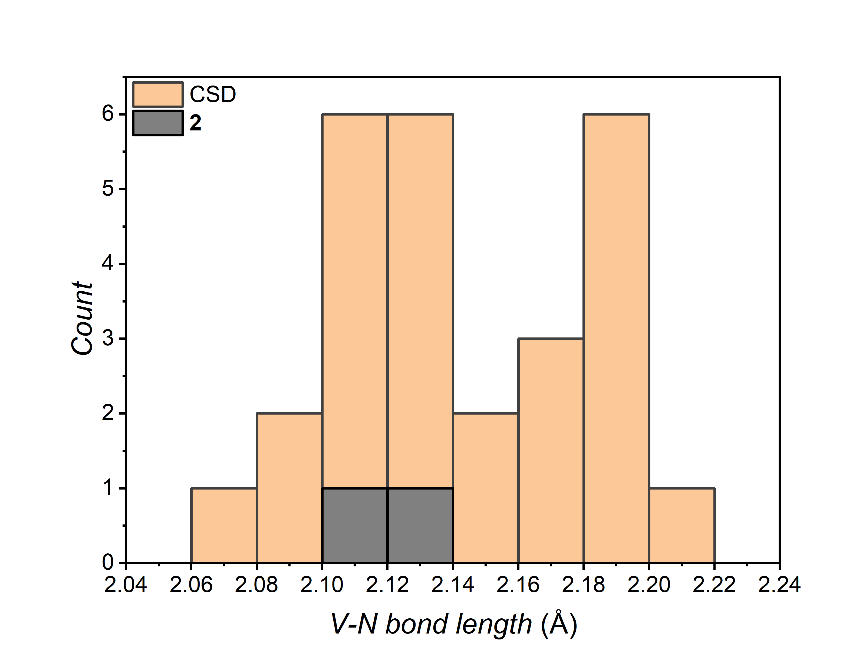


**Figure S2.** Histogram of V-N bond lengths in vanadium(II) complexes with six N-donor ligands (Cambridge Structural Database, 10.08.2024), compared with experimental V-N bond lengths observed in **2**.


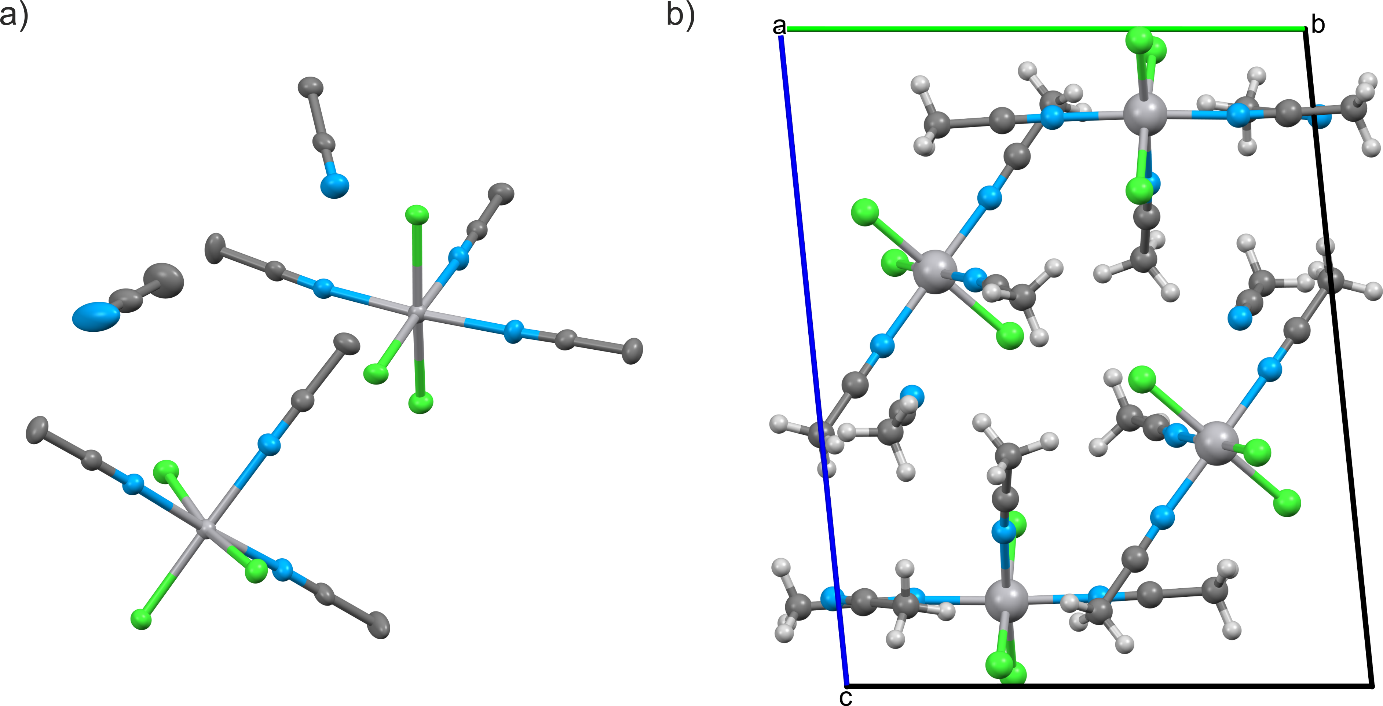


**Figure S3.** a) Asymmetric unit of **3**. Ellipsoids are depicted at 30% probability level, hydrogen atoms were removed for clarity. b) Unit cell of **3** along crystallographic *a* axis. V – grey, Cl – green, N – blue, C – dark grey, H – white.


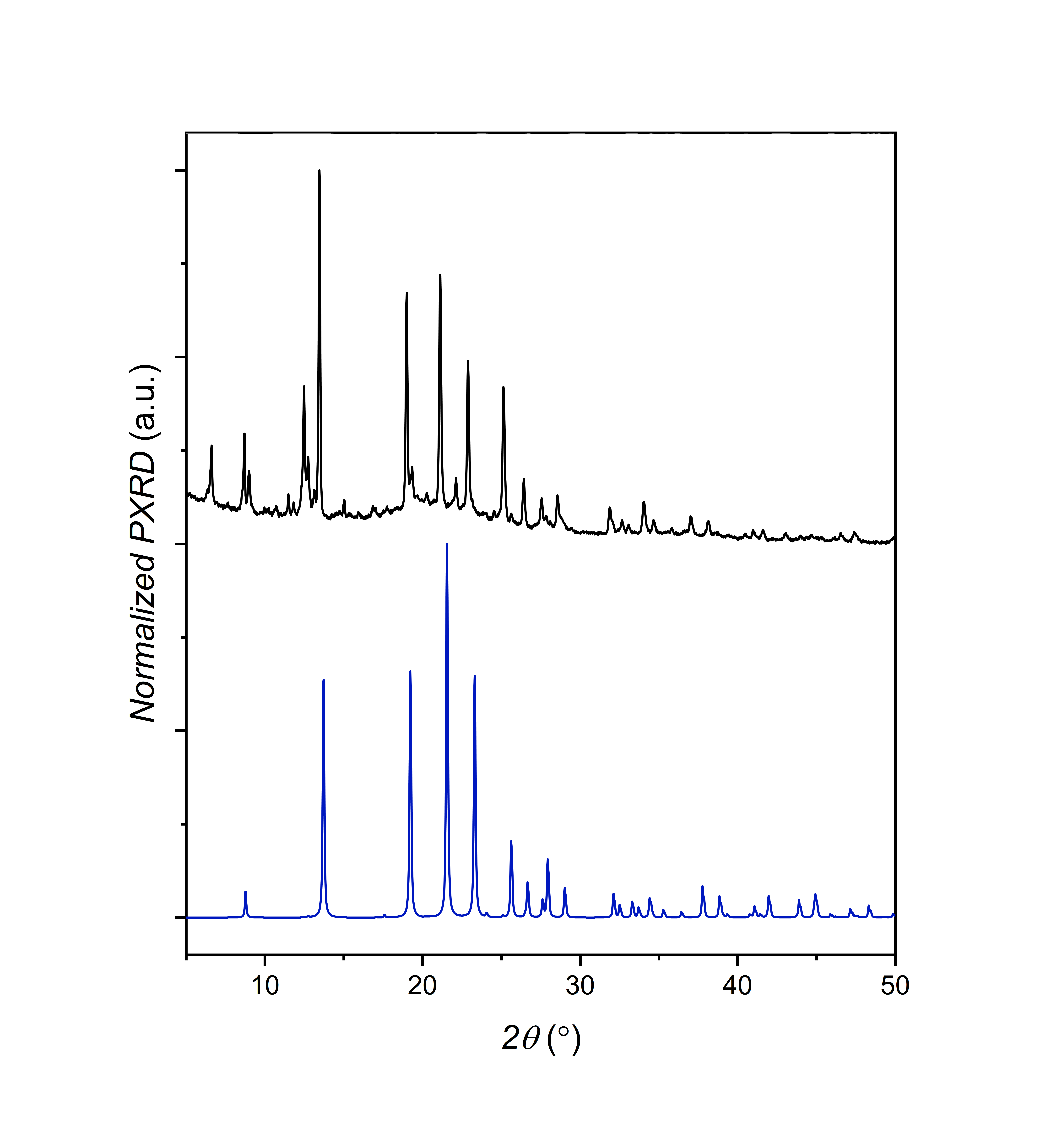


**Figure S4.** PXRD pattern obtained at room temperature immediately after liquid-assisted grinding of [V^II^(CH_3_CN)_6_](BF_4_)_2­_ (**2**) and [K(crypt-222)]_3_[Mo^III^(CN)_6_]·2CH_3_CN (**4**) (black line), and PXRD pattern simulated for the crystal structure of [K(crypt-222)]BF_4_ (blue line; structure code HASVAB in the CSD database^1^).


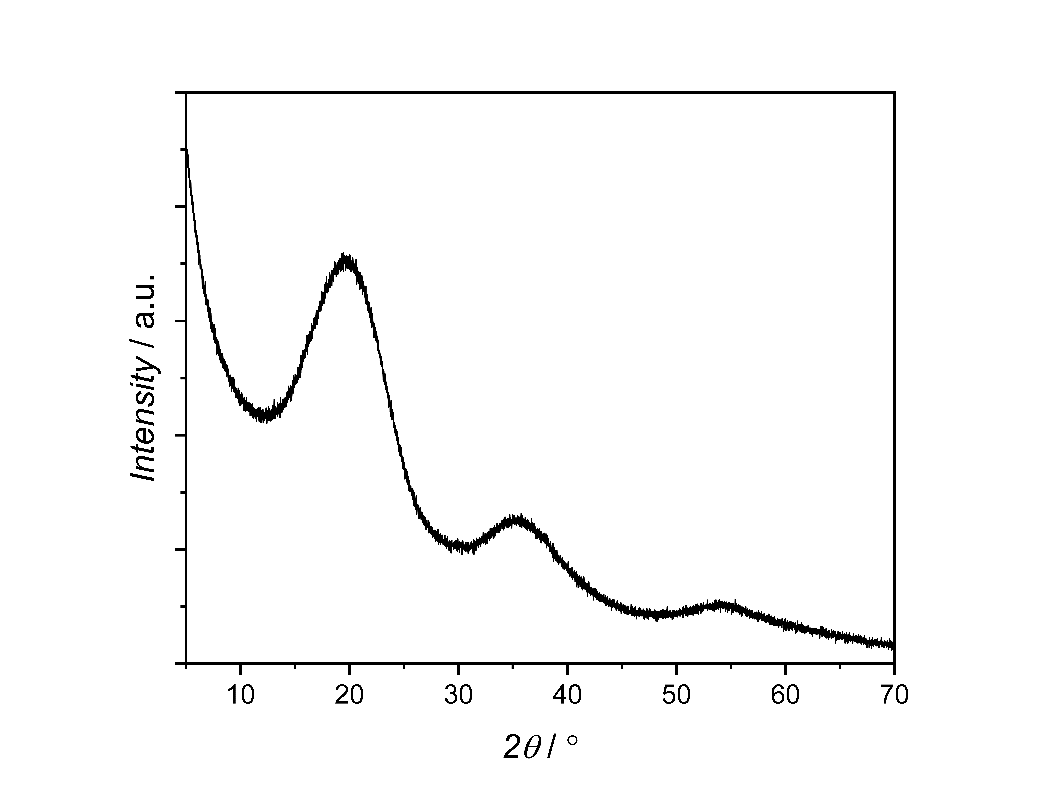


**Figure S5.** PXRD pattern collected for **1** at room temperature.


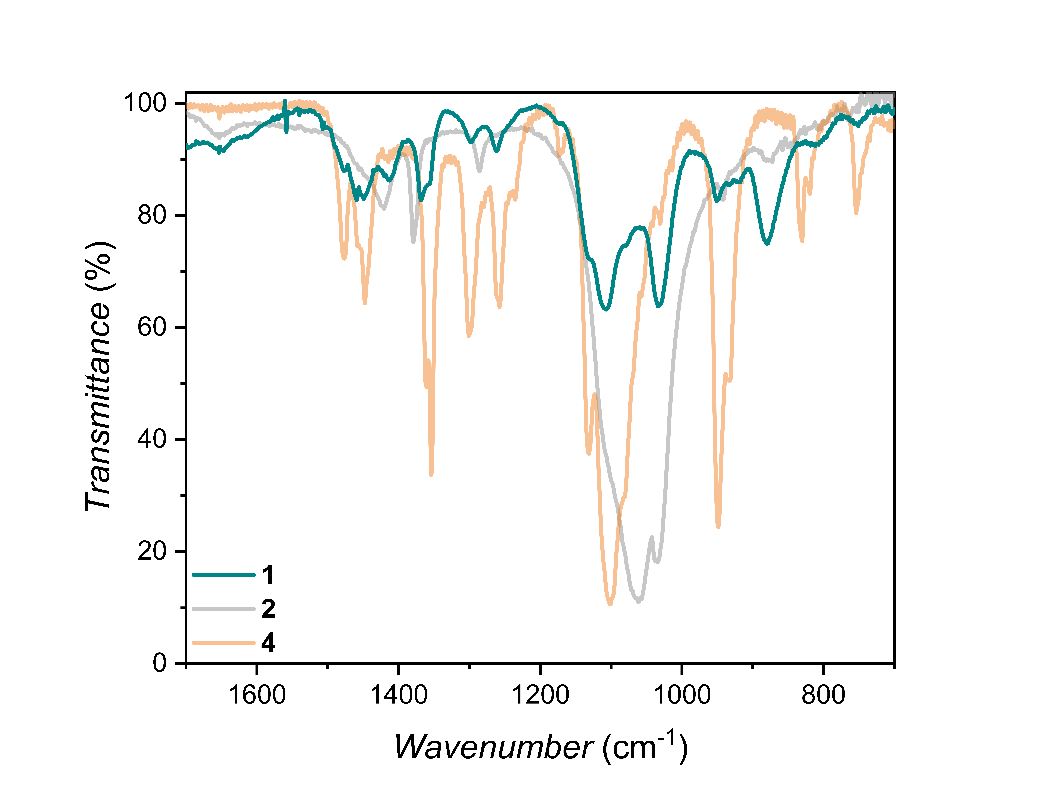


**Figure S6**. Fingerprint region of the room-temperature IR spectrum for **1** (dark green), compared to the IR spectra for precursors **2** (grey) and **4** (orange).


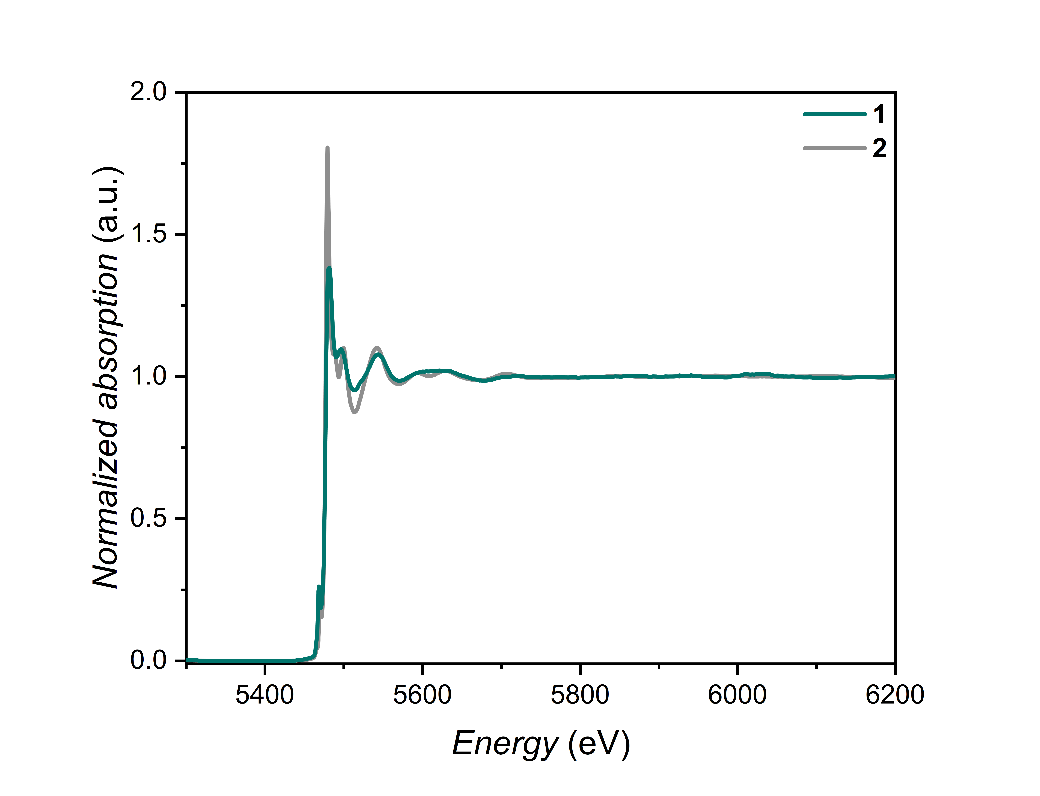


**Figure S7**. Vanadium *K*-edge XAS spectra recorded in the transmission mode at the ASTRA beamline (SOLARIS) for **1** (dark green) and **2** (grey) at room temperature.

**Details of the EXAFS analysis**

The k²-weighted, Fourier-transformed EXAFS spectra uncorrected for phase shift are presented in Figure 2b (main text). The FT spectra provide insight into the local structure and multiple scattering environments of the vanadium centers in these complexes.

To identify the local structures of the samples, or at least define their main scattering paths, V K-edge EXAFS spectra were analyzed. The following parameters were fitted in the analysis:

*S*₀² — amplitude reduction factor;

*R* — path length (radial distance from the first coordination shell);

*σ*² — parameter describing disorder in the system;

Δ*E*₀ — parameter for aligning the energy scale of the theoretical and measured spectra.

S₀² is a non-structural parameter, fitted during the analysis of compound **2**. EXAFS data fitting for the first and second coordination shells was carried out by Fourier transforming the EXAFS spectra in the k range from 2.4 Å⁻¹ to 9 Å⁻¹ (with dk = 0.6). In *R*-space, fits of the Fourier-transformed spectra were performed in the range of *R*_min_ = 1.0 Å to *R*_max_ = 3.2 Å, using theoretical models generated from crystal structure of **2**. Interatomic distances *R*, disorder factors *σ*² for the bonds, and corrections to photoelectron reference energies, Δ*E*₀, were used as fitting parameters. The results of the V K-edge EXAFS data fitting are shown in Figure S8 and Table S2.

From the analysis (Figure S8 and Table S2), it is evident that for **2**, the amplitude at R = 1.66 Å corresponds to V–N bonds, whereas the amplitude at R = 2.42 Å is a superposition from single scattering V–C paths and multiscattering paths presented in Table S2.

From Figure 2b, it is evident that compound **1** — featuring a cyanide bridge (V^II^–NC–Mo^III^) — shows a marked reduction in FT-EXAFS amplitudes across all shells compared to **2**. This attenuation is most pronounced for the main peak near 1.56 Å, arising from the V–N single scattering path, and also affects higher-shell features from V–C–V and V–N–V paths. This diminished amplitude is attributed to several interrelated factors: (1) the heterometallic bridge introduces static disorder at the vanadium site, increasing the Debye–Waller factor (σ²) and damping the EXAFS signal;^2,3^ (2) the bridge perturbs the regularity and coherence of multiscattering pathways, weakening V–N–V and V–C–V contributions relative to a more symmetric nitrogen environment;^4^ and (3) altered backscattering, consistent with electron density extension towards molybdenum, further reduces amplitude in bridge-associated paths.^5^ The FT-EXAFS of **2** reveals not only a robust main peak as compared to **1** but also pronounced, well-resolved higher-shell and multiscattering features, indicating strong multiscattering due to its crystalline character and high symmetry. This agrees with EXAFS theory, which predicts enhanced multiscattering for rigid, highly symmetric environments.

The first-shell V–N peak for **1** appears shifted to a slightly lower apparent *R* as compared to **2**. This shift results from both the phase shift inherent to EXAFS and further modifications by electronic and geometric effects of the cyanide-bridged structure. In such heterometallic systems, altered scattering potentials and coherence introduce larger phase shifts and apparent bond length deviations.^6^

**Table S2**. Best-fit parameters derived from the analysis of the V K-edge EXAFS.

| Path* | *N* [–] | *R_ef_*_f_ [Å] | S_0_^2^ [–] | *ΔE_0_* [eV] | *ΔR* [Å] | *σ^2^* [-] | *R-factor [%]* |
| --- | --- | --- | --- | --- | --- | --- | --- |
| V-N3-V, *ss* | 6 | 2.119 | 0.73±0.13 | 2.6 ±1.8 | 0.008±0.018 | 0.0032±0.0027 | 1.6 |
| V-C4-V, *ss* | 6 | 3.236 |  |  | 0.061±0.021 | 0.001±0.003 |  |
| V-N3-C4-V, fs | 8 | 3.237 |  |  | *ΔR_N3_+ ΔR_C4_* | *σ^2^_N3_+ σ^2^_C4_* |  |
| V-N3-C4-N3-V,df | 4 | 3.238 |  |  | *ΔR_N3_+ ΔR_C4_ +ΔR_N3_* | *σ^2^_N3_+ σ^2^_C4_ + σ^2^_N3_* |  |
| V-N2-C6-V, ft | 4 | 3.241 |  |  | *ΔR_N3_+ ΔR_C4_* | *σ^2^_N3_+ σ^2^_C4_* |  |
| V-N2-C6-N2-V, ft | 2 | 3.245 |  |  | *ΔR_N3_+ ΔR_C4_ +ΔR_N3_* | *σ^2^_N3_+ σ^2^_C4_ + σ^2^_N3_* |  |

* ss – single scattering, fs – forward scattering, df – double forward, ft – forward triangle


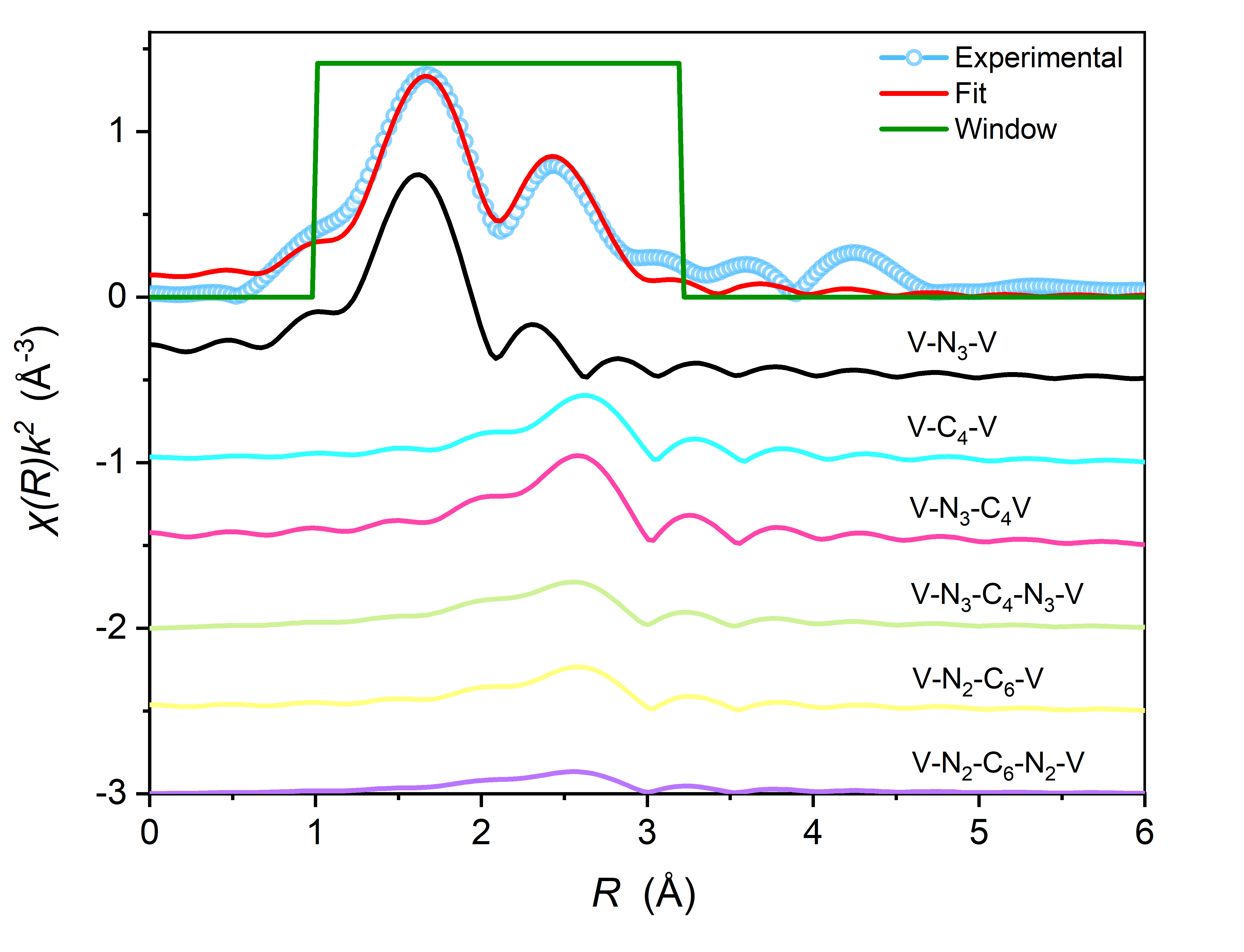


**Figure S8.** Fourier transform of *k*²-weighted *χ*(*k*) (FT-EXAFS) with distinct scattering path contributions for **2**.


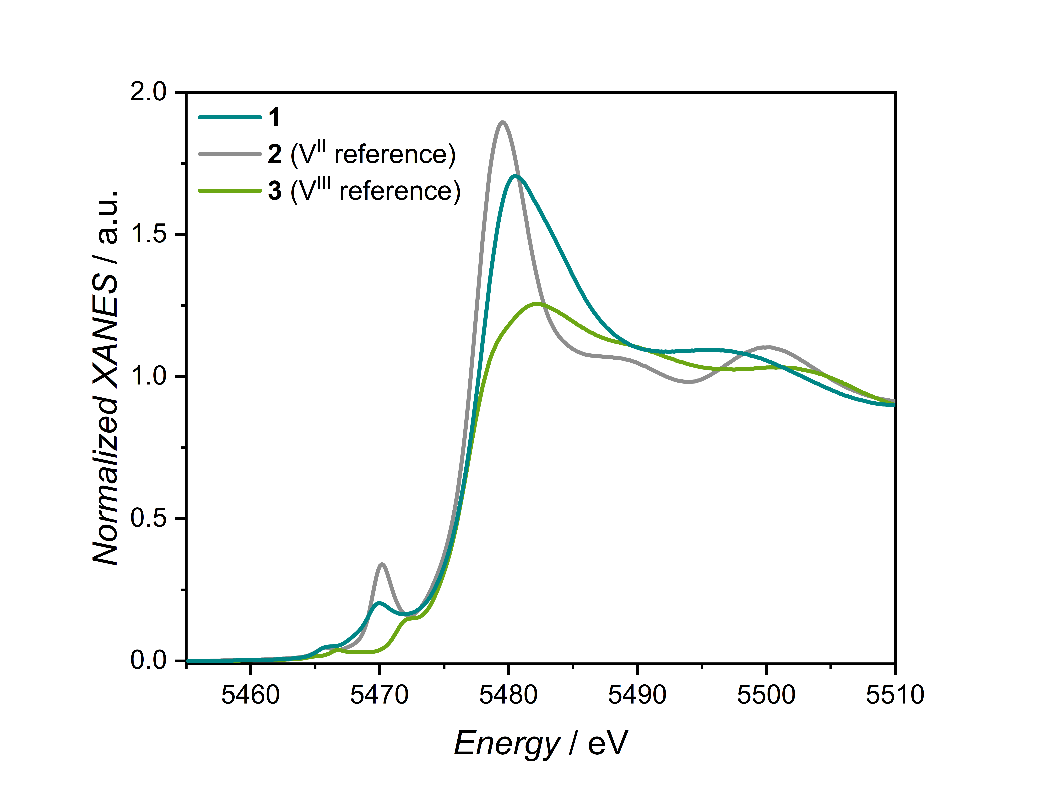


**Figure S9.** Vanadium *K*-edge XAS spectra recorded in the total fluorescence yield detection mode at the ID12 beamline (ESRF) for **1** (dark green), **2** (grey) and **3** (light green) at room temperature.

**
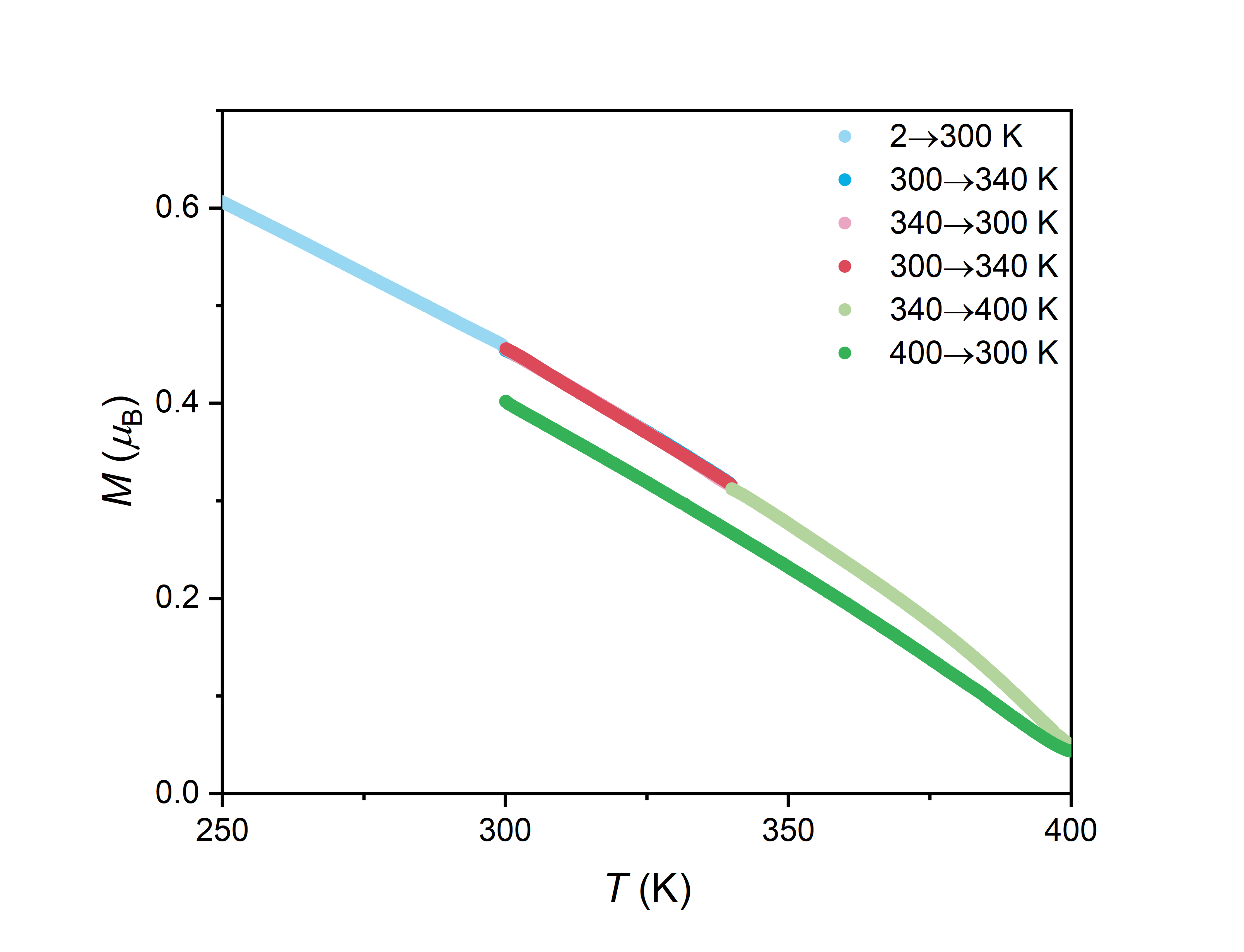
**

**Figure S10.** Magnetization versus temperature curves recorded for **1** in subsequent heating and cooling cycles at 2 K·min^-1^ temperature sweep rate (*μ*_0_*H* = 0.1 T).


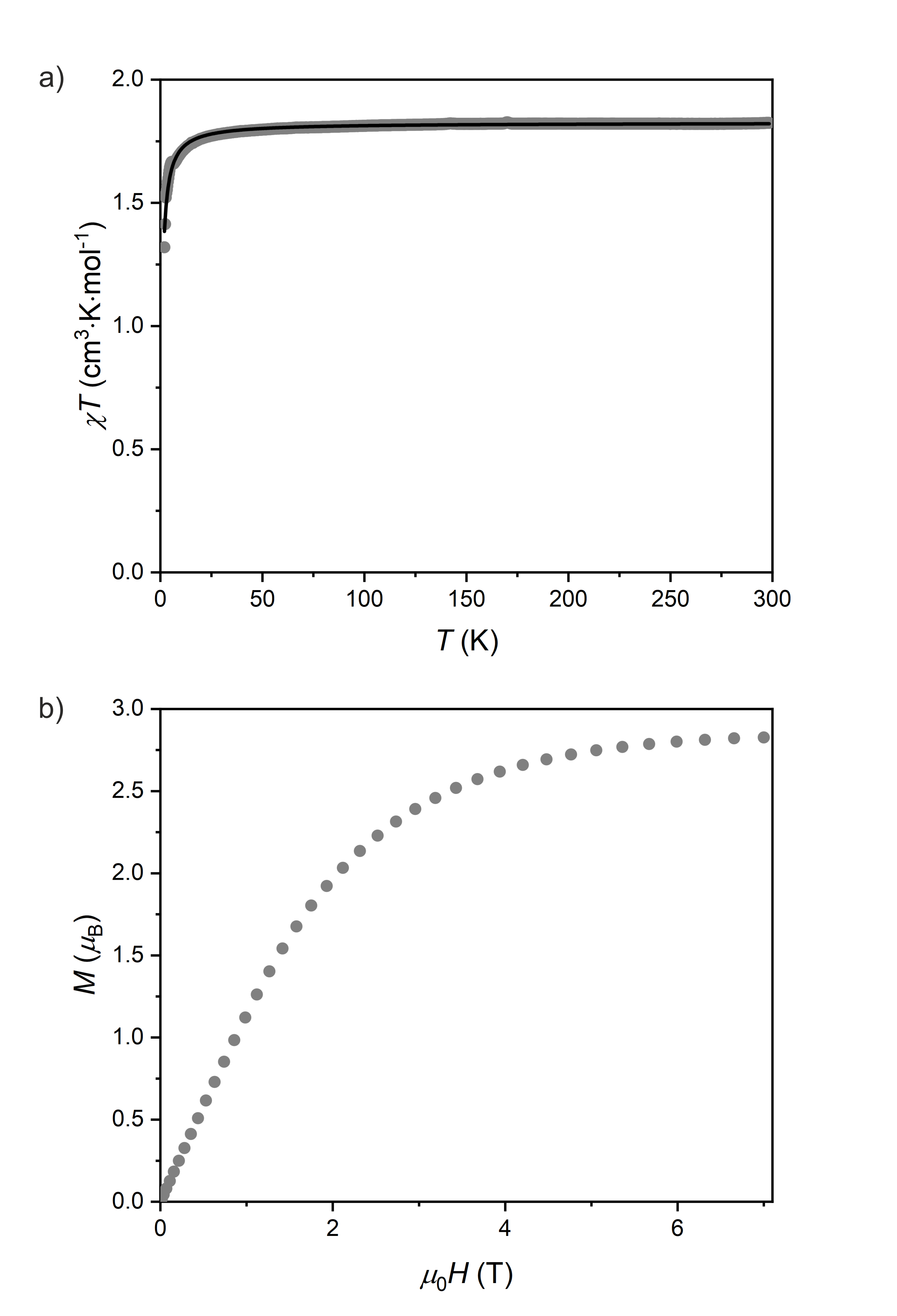


**Figure S11**. a) Thermal dependence of magnetic susceptibility (*χ*) and temperature product for **2** (grey points) and Curie-Weiss fit (black line; *S* = 3/2, *g* = 1.97 and *θ* = -0.64 K). b) Magnetization versus field curve recorded for **2** at 2 K.


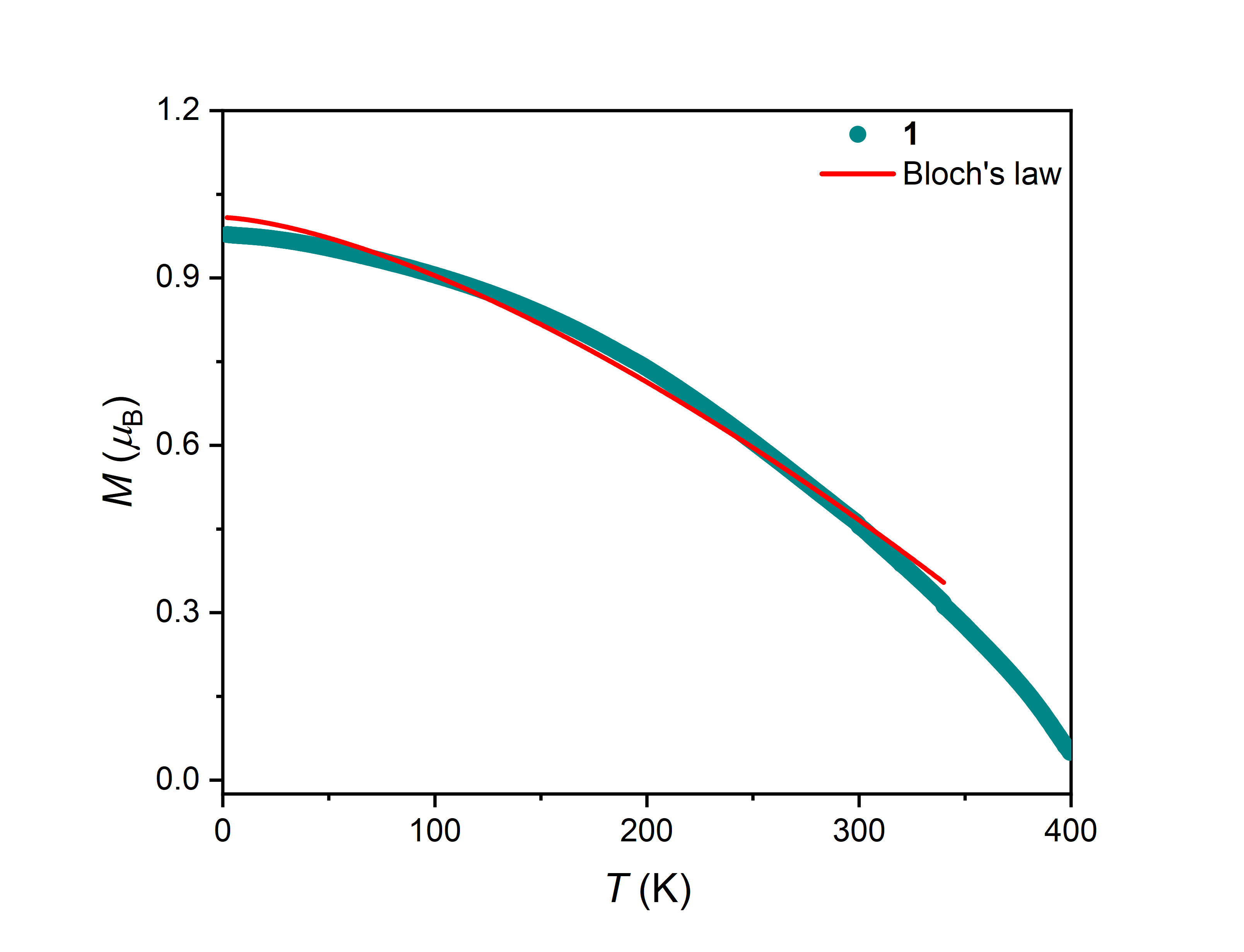


**Figure S12.** Magnetization versus temperature curve recorded for **1** on heating (green line; *μ*_0_*H* = 0.1 T) and fit of the data in the 2-340 K range to the Bloch’s *T*^3/2^ law (*M*(*T*) = *M*_0_·[1-(*T*/*T*_c_)^3/2^]; red line).


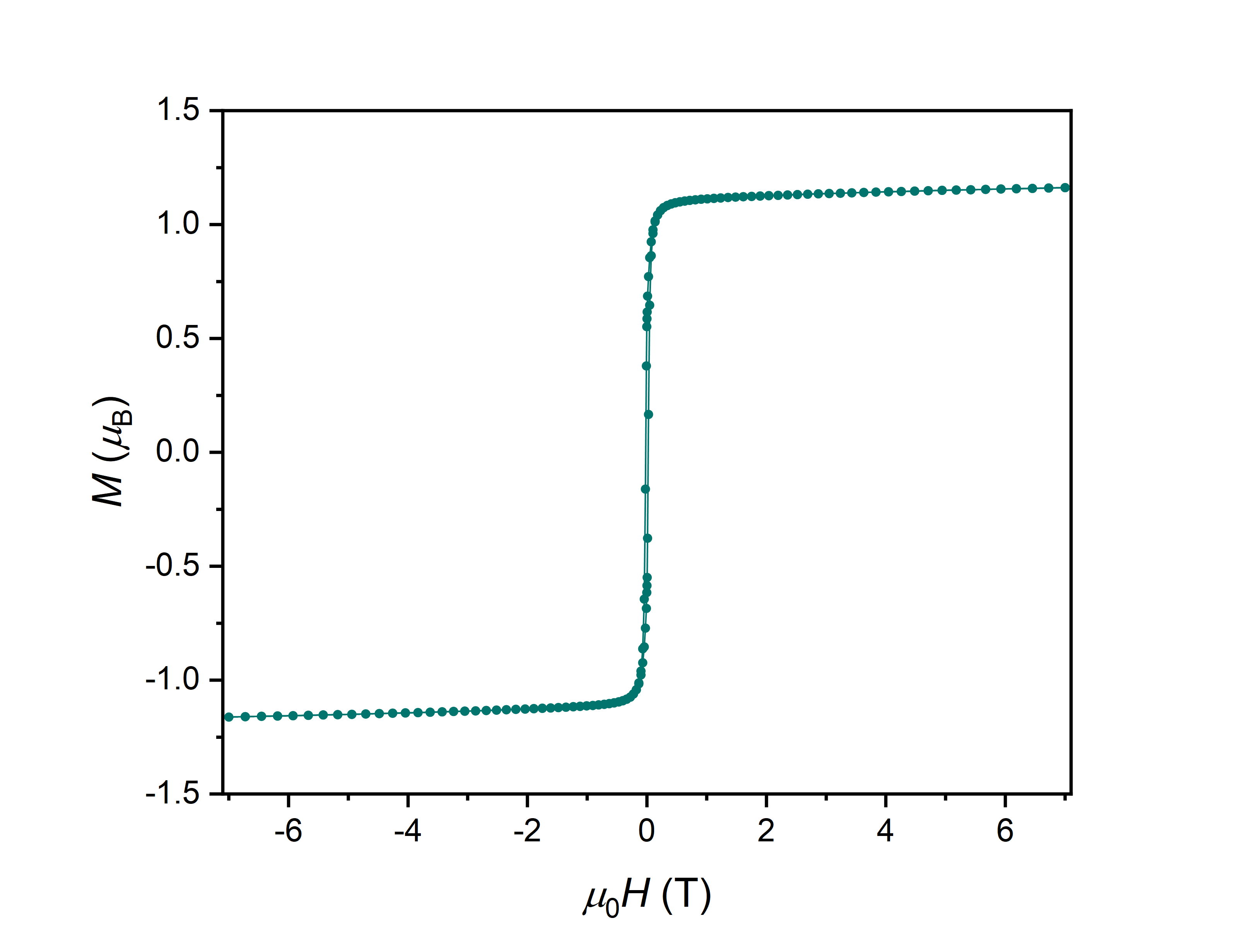


**Figure S13**. Magnetization versus field curve recorded for **1** at 2 K.


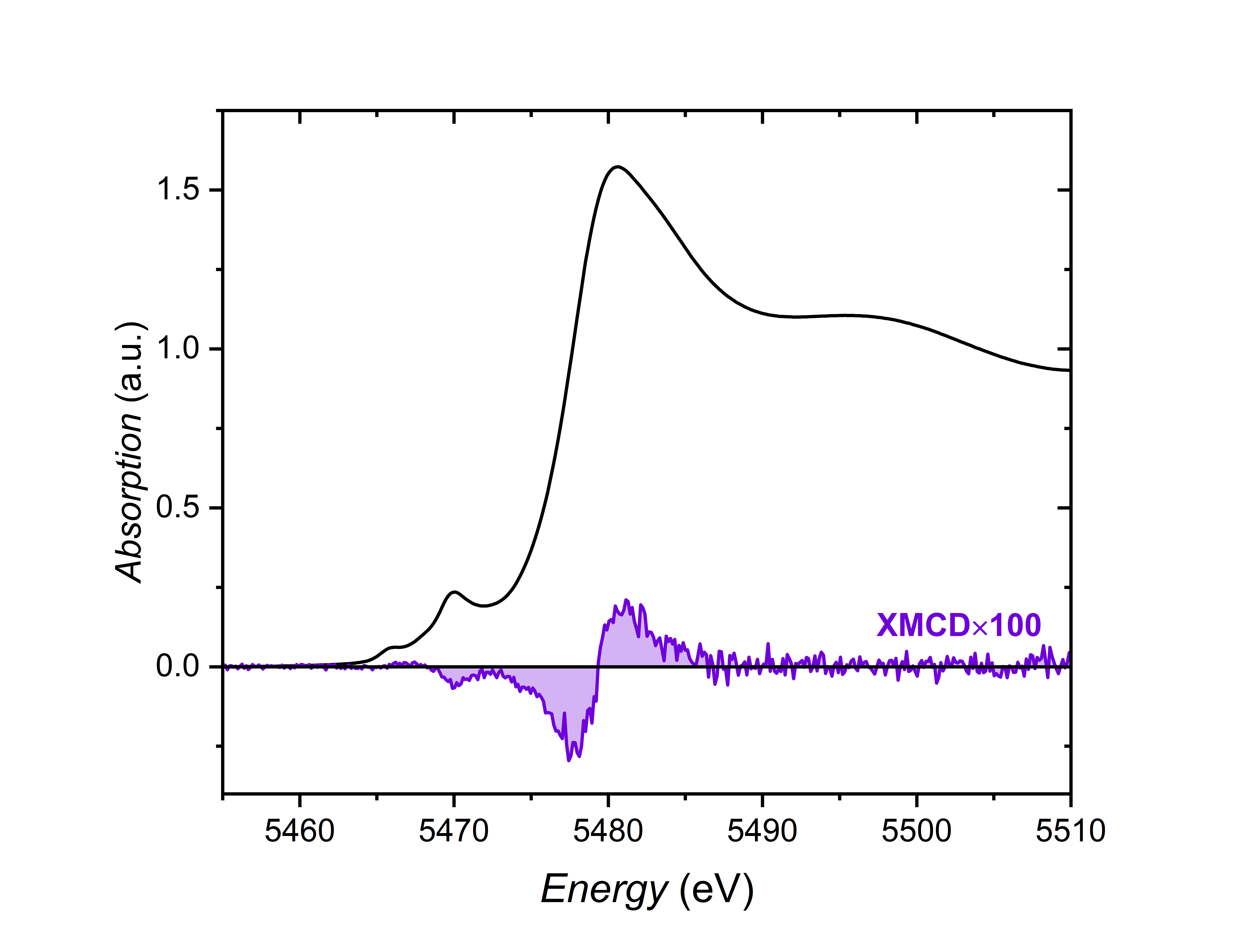


**Figure S14**. Room temperature XAS spectrum recorded in the total fluorescence yield detection mode at the vanadium *K*-edge for **1** (black line), and the corresponding XMCD signal at 295 K (purple line; XMCD spectrum was multiplied by a factor of 100 for better visibility).


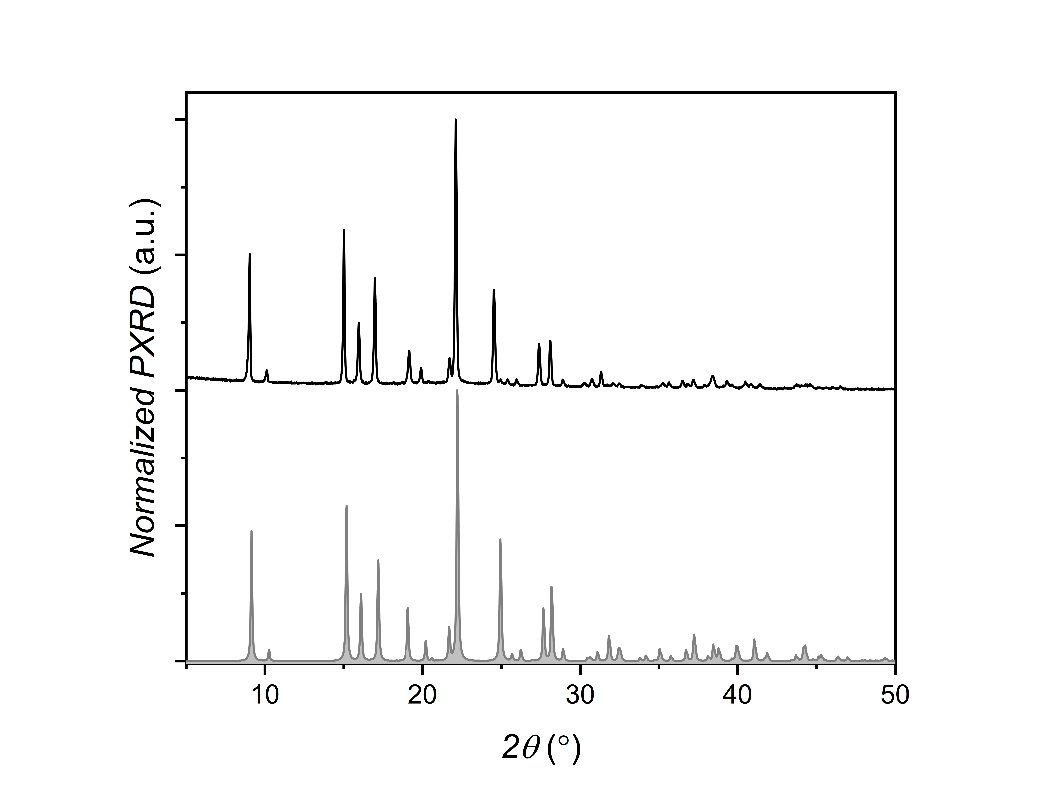


**Figure S15.** Experimental PXRD pattern obtained at room for [V^II^(CH_3_CN)_6_](BF_4_)_2­_ (**2**; black line) and PXRD pattern simulated for the crystal structure at 180 K (grey line).


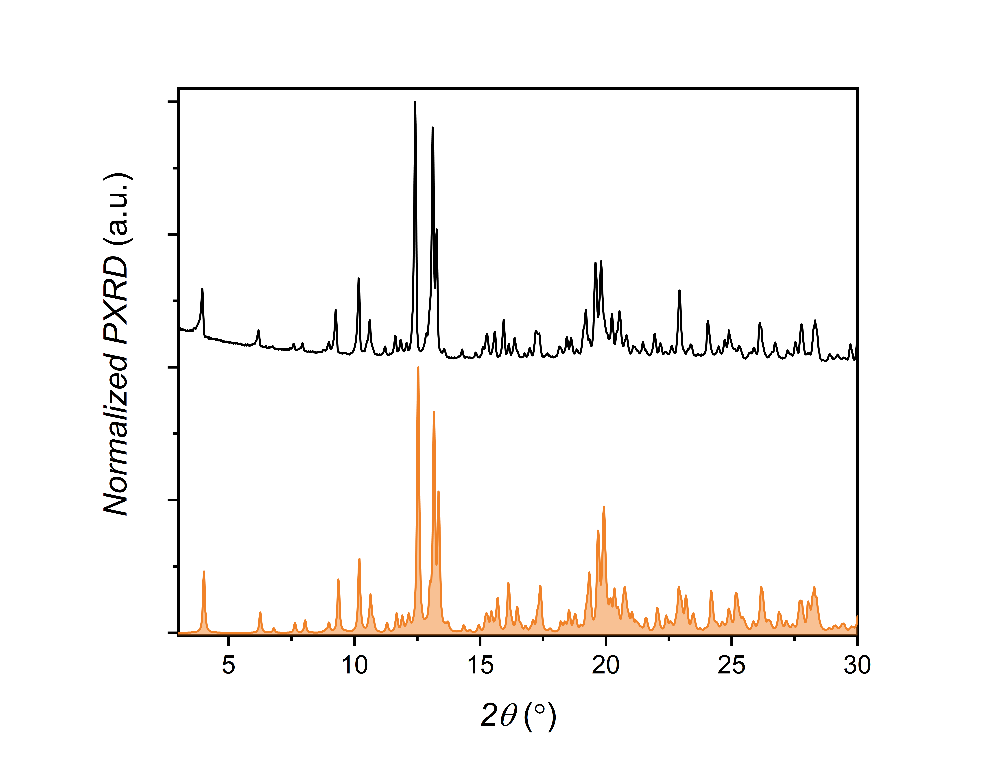


**Figure S16.** Experimental PXRD pattern obtained at room temperature for
[K(crypt-222)]_3_[Mo^III^(CN)_6_]·2CH_3_CN (**4**; black line) and PXRD pattern simulated for the crystal structure at 293 K (orange line).


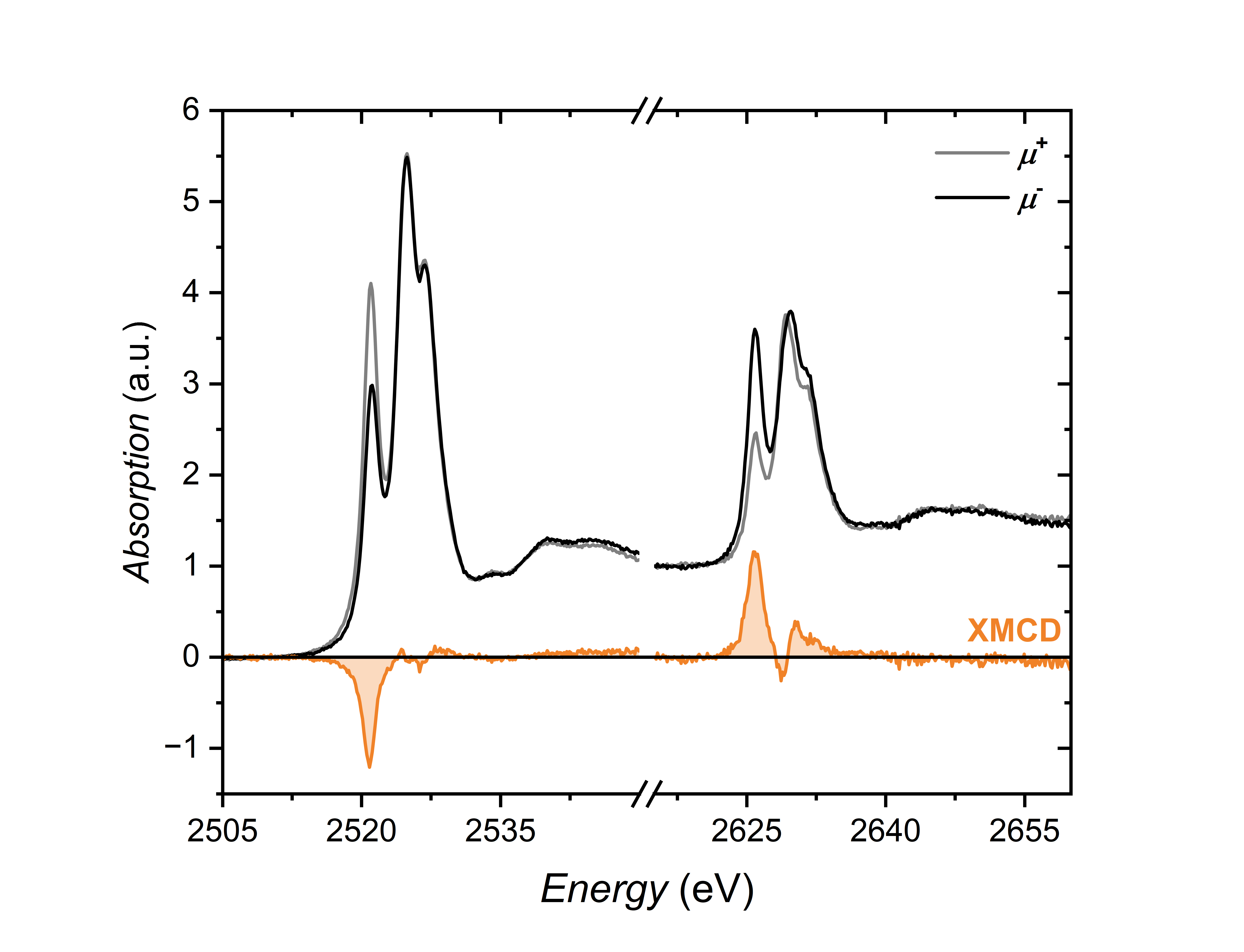


**Figure S17**. Mo *L*_2,3_-edges XANES spectra recorded for **4** at the opposite circular polarizations of incoming X-rays (black and grey lines), and the corresponding XMCD signals (orange lines) at 7 K. The application of the magneto-optical sum rules affords *M*_S,eff_ = 1.30 *μ*_B_, *M*_L_ = -0.06 *μ*_B_ and *T*_z_ = -0.15.


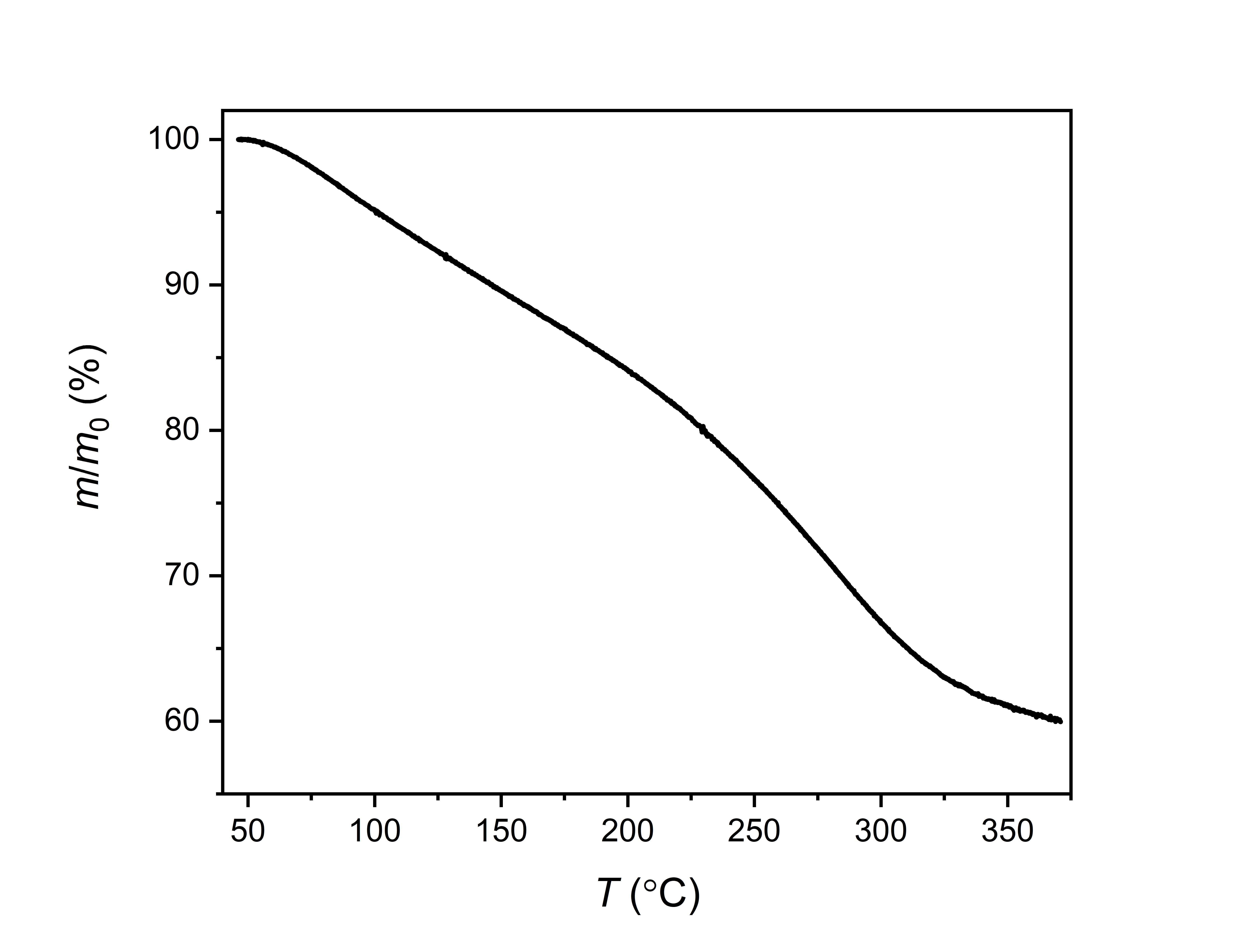


**Figure S18**. TGA recorded for **1** at 2 K·min^-1^ heating rate under dry nitrogen atmosphere.

**References in the SI**

1. Miloserdov, F.M. et al., The Trifluoromethyl Anion: Evidence for [K(crypt-222)]^+^. *Helv. Chim. Acta* **100**, e1700032 (2017)

2. van Bokhoven, J. A. et al., Extended X-ray Absorption Fine Structure Analysis in Catalysis. In: *Modern Coordination Chemistry*. Springer 2002, Ch. 6.

3. Zhang et al., *J. Am. Chem. Soc.* **119**, 2470 –2478 (1997)

4. Sung Co, M. et al., Multiple-scattering effects in EXAFS spectroscopy of oxygen-bridged iron complexes. Possibility of angle determination of EXAFS analysis, *J. Am. Chem. Soc.* **105**, 1144–1150 (1983)

5. Endicott, J. F. et al., Electronic coupling between metal ions in cyanide-bridged ground state and excited state mixed valence complexes, *Coord. Chem. Rev.* **257**, 1676-1698 (2013)

6. Murari, A. et al., Phase shift studies and bond length determination of some copper systems, *Phys. Scr.*, **50**, 423-426 (1994)
